# The structure and functions of TRAF families and recent advances of TRAFs in leukemia

**DOI:** 10.3389/fonc.2026.1738210

**Published:** 2026-02-25

**Authors:** Le Gao, Meimei Yan, Ruochen Xu, Linping Xu, Yongping Song, Wei Li, Xudong Li

**Affiliations:** 1 The Affiliated Cancer Hospital of Zhengzhou University & Henan Cancer Hospital, Zhengzhou, China; 2 Department of Hematology, The First Affiliated Hospital of Zhengzhou University, Zhengzhou, Henan, China

**Keywords:** leukemia, NF-κB, signaling pathways, TRAF6, TRAFs

## Abstract

Leukemia is a malignant clonal disease of hematopoietic stem cells. Currently, primary treatments include chemotherapy, targeted drugs, hematopoietic stem cell transplantation, and immunotherapy. Although advances in treatment have improved survival for leukemia patients, treatment failure still occurs in some individuals for various reasons. This necessitates the discovery of new pathogenic mechanisms and the exploration of novel biological targets. Tumor Necrosis Factor Receptor-Associated Factors (TRAFs) are a family of cytoplasmic adapter proteins, typically comprising seven members. The TRAF protein family is widely involved in cell proliferation, differentiation, survival, and apoptosis, and also regulates immune and inflammatory responses. TRAFs perform dual roles in a broad range of biological activities—as adapter proteins and as E3 ubiquitin ligases—both essential for activating receptor-mediated signaling. In recent years, growing evidence has highlighted the significant role of TRAFs in leukemia, linking them to leukemic stem cell activity, drug resistance, apoptosis, and autophagy. This review introduces the functions and characteristics of TRAFs and summarizes research progress on their involvement in leukemia, underscoring their potential as novel therapeutic targets for the disease.

## Highlights

- The crucial role of the TRAF family members in leukemia is highlighted.
- TRAFs modulate leukemia development by regulating key signaling pathways (NF-κB, PI3K/AKT, WNT) and mediating ubiquitination-dependent degradation of substrates (ULK1, MCL1, p65, MYC).
- Inhibiting TRAF expression disrupts the NF-κB signaling pathway, thereby modulating leukemic cell activity.
- TRAF family members are promising target for leukemia treatments.

## 1 Background

Leukemia is a malignant disease of the hematopoietic system, accompanied by abnormal or underdeveloped blood cells accumulated in the blood, bone marrow and lymphatic system, with anemia, bleeding, infection and other symptoms. Traditionally, according to the speed of onset and the lineage of origin cells, leukemia can be divided into acute lymphoblastic leukemia, acute myeloid leukemia, chronic lymphocytic leukemia and chronic myeloid leukemia (1–6). At present, the main treatments of leukemia are chemotherapy, targeted drugs and hematopoietic stem cell transplantation(HSCT), and some patients can use radiotherapy and immunotherapy. Although a large number of data from basic and clinical trials provide better evidence for new targeted drugs, CAR-T cell therapy and some combination therapies. However, due to the complexity and individual heterogeneity of leukemia, the treatment of patients has not achieved satisfactory results, and prognosis remains poor (7–11). It is necessary to explore the pathogenesis of leukemia, the emergence of drug resistance and explore new therapeutic targets (12–15).

Tumor necrosis factor receptor (TNFR)-associated factors (TRAFs) is a family of proteins with important signal regulatory functions, which are widely involved in biological activities such as cell growth, proliferation, apoptosis, immunity and inflammation (16–18). The TRAF family also plays an important role in tumors, and different members play a role in promoting or inhibiting cancer in different tumors (19–22). Most of the members of the TRAF family have typical structures, including C-terminal TRAF domain, N-terminal RING finger domain and zinc finger motifs. Similar structural characteristics determine their common function, that is, mediating intracellular signal transduction and acting as E3 ubiquitin ligase (23, 24). In recent years, accumulating evidence have confirmed that members of the TRAF family play an important role in leukemia and are expected to become new therapeutic targets for leukemia. This paper describes the main characteristics of the TRAF family, introduces the functions and related signal pathways of the members of the TRAF family, especially TRAF6, and focuses on their related research progress in leukemia.

Given the emerging importance of TRAF proteins in leukemia pathogenesis, a comprehensive understanding of their structural organization is essential for elucidating their functional mechanisms and identifying potential therapeutic vulnerabilities. In the following section, we systematically examine the molecular architecture of TRAF family members, which provides the structural foundation for their dual roles as adaptor proteins and E3 ubiquitin ligases.

## 2 Composition and structural characteristics of TRAF families

Tumor necrosis factor receptor (TNFR)-related factors (TRAFs)are a class of adaptor proteins found in the cytoplasm in 1994. At present, it mainly includes seven members (6 classical members TRAF1–6 and 1 non-classical members TRAF7). The carboxyl terminal of TRAF1–6 contains a common amino acid fragment, namely TRAF domain, which has also become a defining feature of the TRAF family (25, 26).

**Figure 1:**
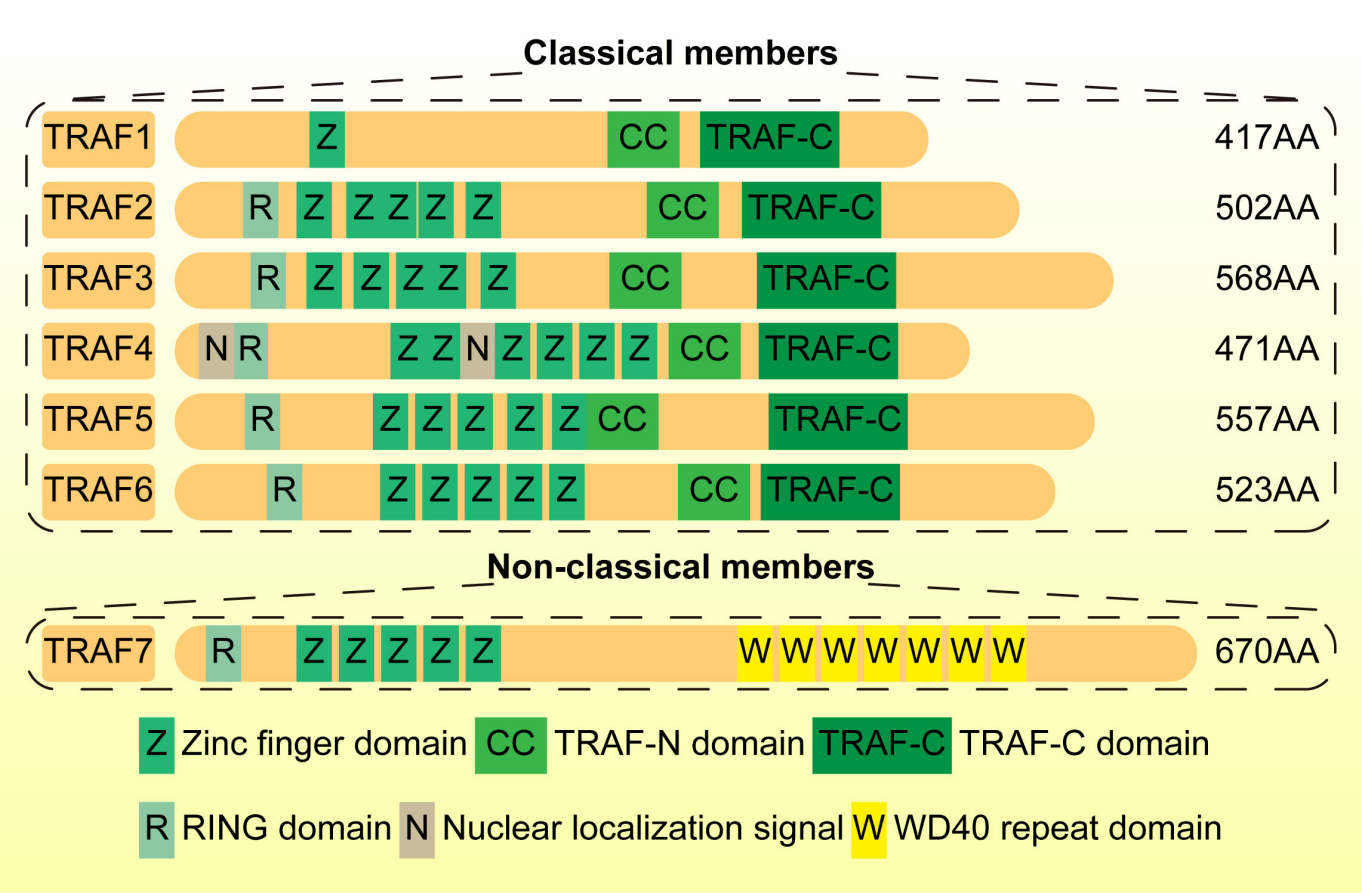
The structure of TRAF family members. TRAFs mainly include seven members (6 classical members TRAF1–6 and 1 non-classical members TRAF7). The carboxyl terminal of TRAF1–6 contains a common amino acid fragment, namely TRAF domain, which has also become a defining feature of the TRAF family.

Analyzing the structural characteristics of TRAF family members facilitates understanding of their functions. TRAF domain can be divided into an amino-terminal coiled-coil region (TRAF-N) and a C-terminal domain (TRAF-C). The former controls the homologous trimerization of TRAF, while the latter can promote TRAF oligomerization and promote the interaction with upstream regulatory factors. There are a wide range of upstream regulatory factors that interact with the TRAF-C terminal, including receptor intracellular domain (TNFR2,CD40 and BAFF receptor), intermediate adaptor proteins (TNFR1-associated death domain protein (TRADD)) and members of the IL-1R-associated kinase (IRAK) family (involved in Toll-like receptor (TLR) and interleukin-1 receptor (IL-1R) signaling) (27, 28). TRAF-N and TRAF-C can also combine with downstream effectors. For example, the TRAF-N domain of TRAF2 can bind to cellular inhibitor of apoptosis 1 (cIAP) and the TRAF-C domain of TRAF3 can bind to nuclear factor-κB (NF-κB)-inducing kinase (NIK). These interactions are essential for the regulation of downstream signaling pathways (29–33). In addition, except for TRAF1, the N-terminal domain of most TRAF contains several zinc finger domains and a really interesting new gene (RING) motif, indicating that they have the functional characteristics of E3 ubiquitin ligase (34) (**Figure 1**).

These structural features directly determine the functional versatility of TRAF family members. Understanding how these structural elements translate into specific biological activities is crucial for appreciating their roles in leukemia. We next examine the functional mechanisms through which TRAFs execute their diverse cellular functions.

## 3 The functions of TRAF families

After understanding the structural characteristics of the TRAF family, it is not difficult to find that the TRAF family plays two roles in a wide range of biological activities: adaptor proteins and E3 ubiquitin ligases. Firstly, as adaptor proteins, TRAFs is an intracellular signal molecule that participates in the signal transduction pathways of TNFR family, TLR family, IL-1R family and RIG-I-like receptor (RLR) family members and plays a key role (24). TRAF1 and TRAF2 are the first identified adapter proteins in the TNFR signaling pathway. They bind to activation-dependent receptors and recruit cIAP that make up the related TRAF-binding proteins. Since then, five other members have been shown to play a role in the TNFR signaling pathway. Subsequent studies have gradually confirmed that TRAF proteins can also participate in signal transduction through TLRs and IL-1R family members, and play a key role in the activation of NF- κB and mitogen activated protein kinase (MAPK) pathways (35–38). Secondly, TRAFs also acts as an E3 ubiquitin ligase. It has been confirmed that TRAFs can only mediate the production of K63-linked polyubiquitin chain by cooperating with E2 ubiquitin- conjugating enzyme UBE2D3 (also known as UBCH5C) and UBC13-UEV1A (also known as UBE2N-UBE2V1) (39, 40). This non-degradative protein ubiquitination is essential for the activation of downstream signaling pathways. Studies have shown that TRAF6-mediated K63-linked polyubiquitination is essential for the activation of NF- κB and MAPKs pathways (39, 41). TRAF2 becomes a highly active K63-linked ubiquitin ligase after the binding of sphingosine-1-phosphate (S1P) to its RING finger domain, which ubiquitinates protein kinase receptor interacting protein 1 (RIP1;, also known as RIPK1) and participates in TNFR-mediated signal transduction (42–44). In addition, TRAF2 can also mediate the polyubiquitination of cIAP (45, 46).

In summary, the two functional modes of TRAFs (as adaptor proteins and E3 ubiquitin ligases) are essential for activating receptor-mediated signaling. Most of the time, these two functions can coordinate with each other. TRAFs acts as a junction protein by automatically ubiquitination, exposing ubiquitin binding motifs, binding and activating downstream kinases through exposed sequences, thereby affecting signal transduction (24).

Having established that TRAF proteins function through coordinated mechanisms as both adaptor proteins and E3 ubiquitin ligases, we now turn our attention to current research progress on individual TRAF family members in different leukemia types, highlighting their distinct yet interconnected roles in disease initiation, progression, and therapeutic resistance. We organize this discussion by individual TRAF members (TRAF1-7), with particular emphasis on TRAF6 given its extensively characterized involvement across multiple leukemia subtypes.

## 4 Recent advances and researches of TRAF families in leukemia

### 4.1 TRAF1

TRAF1 is a survival-promoting aptamer molecule in the TNFR superfamily (TNFRSF) signal transduction pathway. It is overexpressed in many B-cell leukemia, including refractory chronic lymphoblastic leukemia (CLL). Edilova et al. found that protein kinase C-related protein kinase N1 (PKN1) plays an important role in protecting TRAF1 from CIAP-mediated degradation during CD40 signal transduction in lymphoma. By screening a library containing 700 kinase inhibitors, they identified two PKN1 inhibitors, OTSSP167 and XL-228, which can induce a decrease in the expression of TRAF1 in patients’ CLL cells, which in turn induces cell death (47). In another CLL study, Xiao et al. found that HuR protein has great potential in CLL therapy. HuR can directly bind to TRAF1, inhibit the down-regulation of TRAF1 expression by HuR protein, promote inflammatory response and apoptosis, and increase the sensitivity of CLL cells to drugs, suggesting that targeted HuR-TRAF1 may provide a new strategy for solving the problem of drug resistance in CLL (48).

TRAF1 is a NF- kappa B inducible protein (49). Many natural extracts can inhibit the activity of NF- κB and down-regulate the expression of TRAF1, so as to exert the effect of anti-leukemia. Indole-3-carbinol can nonspecifically inhibit the activity of NF- κB, down-regulate downstream genes such as TRAF1, and promote the death of myeloid and leukemic cells (50). C-28methylesterof2-cyano-3,12-dioxoolean-1,9-dien-28-oicacid (CDDO-Me), a synthetic compound, can inhibit NF- κB by inhibiting INBA kinase, thus inhibiting the expression of gene products regulated by NF- κ B, such as TRAF1, and enhancing apoptosis of leukemic cells (51). Gambogic acid (GA) can bind to transferrin receptor, inhibit NF- κ B signal pathway, down-regulate the expression of TRAF1 and other genes, and promote leukemic cell death (52).

### 4.2 TRAF2

Tumor necrosis factor receptor-associated factor 2 (TRAF2) is a dual-functional protein. As a connective protein and ubiquitin E3 ligase, it plays an important role in mediating TNF α-NF κB signaling pathway (53, 54). Violacein is a pigment isolated from purple bacteria in the Amazon River. it has a variety of biological characteristics and can fight leukemia. Ferreira et al. found that violacein induces apoptosis in HL60 cells by affecting the interaction between TRAF2 and tumor necrosis factor receptor 1 signaling (55). KC-53 is a new type of biyouyanagin analogue with strong anti-inflammatory and antiviral activities. Christina et al. found that HL-60 and CCRF/CEM cell lines are the most sensitive to the anti-proliferation effect of KC-53. Mechanism studies have shown that KC-53 can significantly inhibit the serine phosphorylation of TRAF2 and IκBα induced by TNF α, thus inhibiting the nuclear translocation of p65/NF-κB, reducing the transcriptional expression of pro-inflammatory and pro-survival p65 target genes (56). Zinc finger CCCH containing 15 (ZC3H15) is an erythropoietin-induced gene that is expressed in all human tissues (57). Using leukemia gene map (LGA) database, Savva et al. found that the expression of ZC3H15 in AML was significantly higher than that in MDS, CML, ALL and normal bone marrow samples. The results of co-immunoprecipitation showed that there was interaction between ZC3H15 and TRAF2, suggesting that ZC3H15 may participate in NF-κB pathway through TRAF2 and have the potential to become a therapeutic target for AML (58). Leukocyte immunoglobulin-like receptor B (LILRB) is a family of immune checkpoint receptors involved in the occurrence and development of acute myeloid leukemia (AML), but its specific mechanism has not been elucidated (59–61). Wu et al. found that LILRB3 interacts with TRAF2. After LILRB3 is activated, it recruits cFlIP and then activates NF-κB signal pathway to inhibit the effect of anti-tumor T cells. When NK-κB is overactivated, it can interfere with the interaction between TRAF2 and LILRB3 and inhibit the activation of LILRB3 through A20-related negative feedback regulation. Blocking LILRB3 signal can hinder the progress of AML, which confirms that LILRB3 may be a promising therapeutic target for AML (62).

Besides AML, TRAF2, it also plays an important role in other leukemia. Perez-Chacon’s team studied the role of TRAF2 in chronic lymphocytic leukemia/small lymphocytic lymphoma in mice. Their results show that TRAF2 has a significant effect on JNK and ERK activation in response to CD40. However, the absence of TRAF2 reduces BCR-mediated p38 activation without affecting the activation of p38 MAPK by CD40. Further studies have confirmed that TRAF2 is necessary for CD40-mediated cell proliferation, and the deletion of TRAF2 can make B cells survive independent of B cell activating factor, thus promoting the occurrence and development of tumor (63).

Crucially, TRAF2DN/Bcl-2 double-transgenic mice model recapitulates key immunogenetic features of the human CLL. Research indicates that these mice exhibit a biased usage of IGHV gene subgroups and possess stereotyped B-cell receptors (BCRs), suggesting that antigen-specific selection drives the expansion of malignant clones (64). The cooperation between TRAF2 signaling alterations and BCL-2 overexpression is essential for this process, as it enforces marginal zone B cell differentiation and confers resistance to apoptosis, closely mimicking the pathogenesis of human CLL (65).

In CML mouse model, Christian found that CD27, a member of tumor necrosis factor receptor family, was expressed in BCR/ABL+LSCs and leukemic progenitor cells. By binding to its ligand CD70, it promoted the nuclear localization of activated β-catenin, TRAF-2 and NCK- interacting kinase (TNIK), further activated the expression of WNT target gene, and promoted the proliferation and differentiation of LSCs (66). Palumbo study found that autophagy of HAP1 cells increased after knockout of TRAF2. Endoplasmic reticulum stress can mediate JNK activation and autophagy in WT and TRAF2-KO cells, but overexpression of TRAF2C terminal fragments inhibits the response of TRAF2-KO cells to endoplasmic reticulum stress, indicating that TRAF2 plays an essential role in JNK activation and autophagy in HAP1 cells (67).

### 4.3 TRAF3

The study of TRAF3 in leukemia is mainly focused on lymphocytic leukemia. In one study, Pérez-Carretero et al. evaluated a large number of CLL patients with del (14Q) and reported for the first time high frequency changes (deletions and mutations) of TRAF3 bialleles, which have negative clinical significance in CLL patients with 14q32 deletions (68). B cell activating factor (BAFF) is essential for the survival of both healthy B cells and tumor B cells. In the study of CLL, Paiva’s team found that SYK plays an anti-apoptotic role in BAFF-stimulated cells by interacting with the TRAF2/3 complex to mediate the crosstalk of BAFF-B cell receptors (69). In B-ALL, Li et al. found that DYRK1a can interact with TRAF3 and phosphorylate TRAF3 at serine-29, interfering with the role of the latter in mediating Nik degradation, thus promoting BAFF-induced Nik accumulation and atypical NF-kB activation, revealing a new pathogenesis of B-ALL (70).

### 4.4 TRAF4

Tumor necrosis factor receptor related factor 4 (TRAF4) is different from other members of the TRAF family in that it contains a nuclear localization signal (NLS), which plays an important role in ontogeny (71). TRAF4 has E3 ubiquitin ligase activity, promotes ubiquitination of related proteins and regulates downstream signal transduction (72). TRAF4 participates in the transduction of multiple tumor-related signal pathways, which can affect the growth, proliferation, metastasis, apoptosis and drug resistance of breast cancer. In addition, TRAF4 plays an important regulatory role in liver cancer, lung cancer, prostate cancer, colorectal cancer and so on (73). In chronic lymphocytic leukemia (CLL), microRNAs (miRNAs) and its targets can regulate B cell receptor (BCR) signal and T cell interaction, which plays a key role in the pathogenesis and invasion of CLL (74). Through performing complex profiling of short non-coding RNAs, Sharma et al. found that all three members of the miR-29 family (miR-29a, miR-29b, miR-29c) were continuously down-regulated in the immune niche, and low levels of miR-29s corresponded to the high responsiveness of CLL cells to BCR ligation and shorter survival time of CLL patients. Mechanism studies have found that TRAF4 is a new direct target of miR-29s, and the expression of TRAF4 positively regulates the response of CLL to CD40 activation and downstream NF-κB signals. In CLL, BCR inhibitors can attenuate the inhibition of miR-29s by BCR, thus impairing the up-regulation of TRAF4 and the activation of CD40-NF-κB signal. This study found a new miR-29s-TRAF4-CD40 signal axis regulated by BCR, indicating that TRAF4 plays an important role in the pathogenesis of CLL (75).

### 4.5 TRAF5

Studies have confirmed that TRAF5 negatively regulates TLR signaling in B lymphocytes and can also participate in the resistance to oxaliplatin and necrotic ptosis in colorectal cancer (76, 77). At present, the research on TRAF5 in leukemia mainly focuses on acute lymphocytic leukemia (ALL). T-cell acute lymphoblastic leukemia (T-ALL) is an invasive blood cancer that is the most common malignant tumor in children, accounting for 15% of all cases in children (78). Chen et al. studied the role of cyclin-dependent kinase inhibitor 2B anti-sense RNA 1 (CDKN2B-AS1) in the progression and chemotherapy resistance of pediatric T-ALL. Their results show that CDKN2B-AS1 is highly expressed in pediatric T-ALL and is associated with adriamycin resistance. CDKN2B-AS1 deletion has obvious inhibitory effect on T-ALL. The results of downstream mechanism studies show that loss of CDKN2B-AS1 can up-regulate miR-335-3p, while TRAF5 is the direct target of miR-335-3p, TRAF5 mediates the inhibitory regulation of T-ALL by CDKN2B-AS1, and the down-regulation of TRAF5 caused by CDKN2B-AS1 deletion can impair the progression of T-ALL and enhance the sensitivity to doxorubicin (79). In another T-ALL study, Zhou et al. confirmed that the expression level of miR-141-3p in patients was significantly lower than that in healthy controls, while the expression of TRAF5 was opposite to that of miR-141-3p. Mechanism studies have shown that miR-141-3p can inhibit the proliferation of T-ALL cells and promote apoptosis by targeting TRAF5 (80). The above two studies suggest that TRAF5 plays an important role in promoting cancer in T-ALL and may be a potential therapeutic target for T-ALL. In addition, in order to study the possible reasons for the changeable response to treatment in children with B precursor acute lymphoblastic leukemia (ALL), Weston et al. established a DNA damage model by ionizing radiation and found that the expression of TRAF5 increased in anti-apoptotic tumors. Further studies confirmed that the enhancement of NF-κB survival signal caused by overexpression of TRAF5 may be one of the mechanisms of anti-apoptosis in ALL (81).

### 4.6 TRAF7

TRAF7, the last member of the TRAF family, has been found to alter many signal transduction pathways, such as c-Myb, ERK1/2, p53 and NF-κB, which are closely related to tumorigenesis and the development of AML (82). Zou et al. found that TRAF7 is down-expressed in patients with AML and many kinds of myeloid leukemia cells. Overexpression of TRAF7 inhibits the growth of K562 and MOLM-13 cells, promotes apoptosis, impairs the process of glycolysis, and leads to cell cycle arrest in G0/G1 phase. Mechanism studies have shown that TRAF7 affects the glycolysis and cell cycle process of myeloid leukemia cells through the Kruppel-like factor 2 (KLF2) -6-phosphofructo-2-kinase/fructose-2,6-biphosphatase 3 (PFKFB3) axis. In xenotransplantation mice, knocking down TRAF7 can reduce the number of human CD45+ cells in peripheral blood of mice, indicating that TRAF7 can play an anti-leukemia effect (83). In addition, Ding et al. found that miRNA-126 is highly expressed in AML and promotes the development of leukemia by down-regulating TRAF7 and blocking c-flip pathway, which further confirms that TRAF7 plays an important role in leukemia (84).

### 4.7 TRAF6

**Figure 2:**
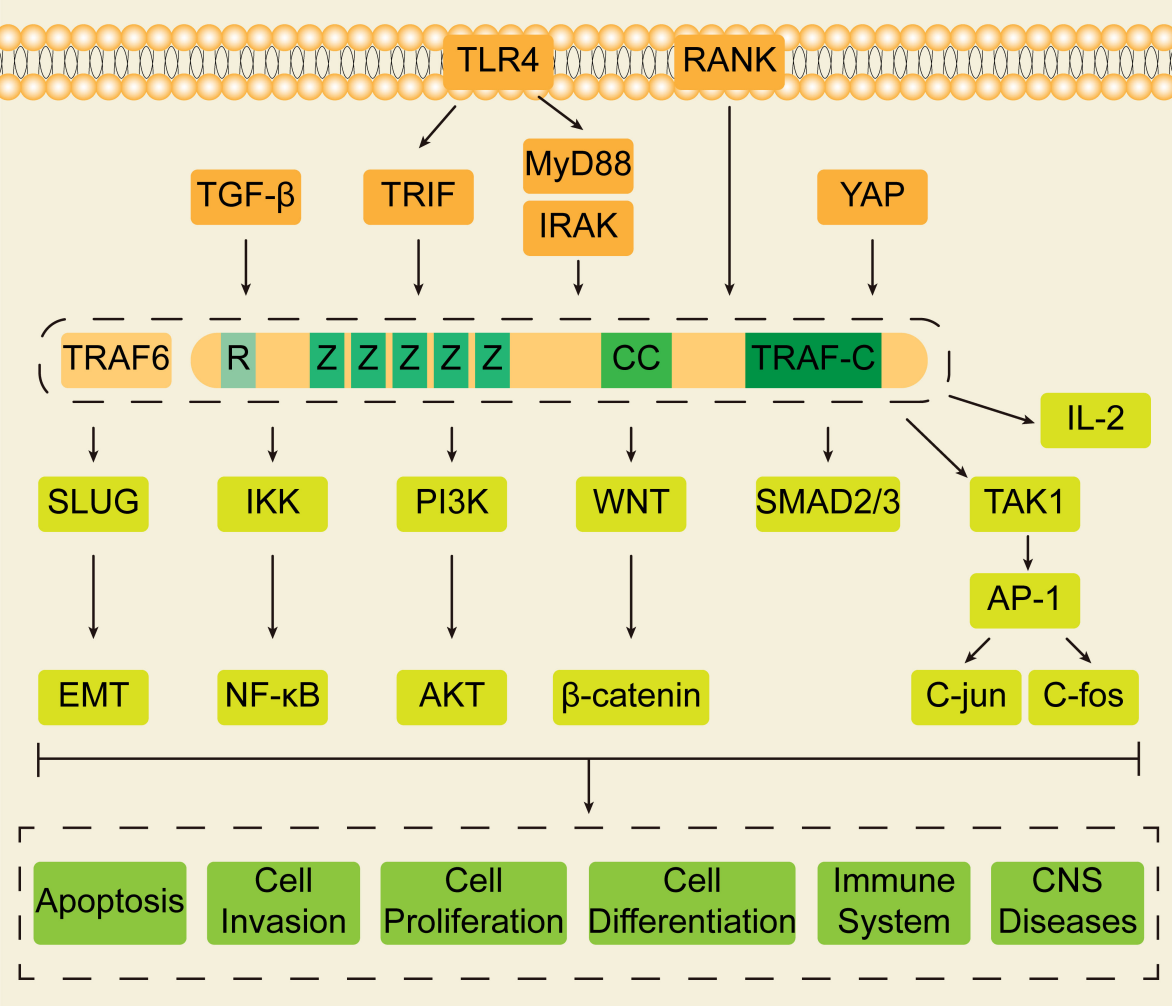
The structure of TRAF6 and related signal pathways. The structure of TRAF6 is similar to that of most members of the TRAF family, the C-terminal contains a TRAF domain composed of a helix and a conservative TRAF-C domain, and the N-terminal contains a ring finger domain and five zinc fingers. TRAF6 is involved in the regulation of multiple signal pathways. TLR4 and RANK are the main upstream signal molecules of TRAF6, in which TLR4 can not only regulate downstream signal molecules by activating TRIF and then recruiting TRAF6, but also promote MyD88 to recruit and activate IRAK, which interacts with TRAF6 and transmits signals. In addition, TGF-β can affect the secretion of IL-2 and the activation of SMAD2/3 through TRAF6, and YAP can inhibit the downstream NF-κB by inhibiting TRAF6. The downstream of TRAF6 is rich in signal pathways, including NF-κB, PI3K/AKT, WNT, EMT, AP-1 and so on. By participating in the above signaling pathways, TRAF6 has important biological functions and can affect apoptosis, cell proliferation, cell differentiation, cell invasion, immune system and central nervous system diseases.

It is known that TRAF6 regulates a variety of biological activities by stimulating the activation of multiple signal pathways (NF-κB,PI3K/AKT,WNT,YAP,TGF-β, etc) (85–90). (**Figure 2**) Also, TRAF6 is widely involved in tumorigenesis, apoptosis, inflammatory response, invasion, migration and so on (91–96). In leukemia, a large number of basic studies have confirmed that these signaling pathways are closely related to the occurrence and development of leukemia, the production of drug resistance, the activity of leukemic stem cells(LSC), leukemic cell proliferation, apoptosis and autophagy and so on (97–101). In AML cells, activated leukocyte immunoglobulin-like receptor B (LILRB) leads to the recruitment of cFlIP and the subsequent upregulation of NF- κB, which increases the survival rate of leukemic cells, inhibits T cell-mediated anti-tumor activity, and thus promotes the development of AML (62). The ectopic expression of TAL1 in about 30% of T cell acute lymphoblastic leukemia (T-ALL) inhibits the growth of T cells by affecting apoptotic genes, while the activation of AKT pathway reduces cell apoptosis and strongly promotes cell proliferation and leukemia *in vivo* (102). WNT signaling pathway is closely related to LSC. Armstrong’s research indicates that WNT/β-catenin signaling pathway is necessary for self-renewal of LSCs derived from hematopoietic stem cells (HSC) or more differentiated granulocyte-macrophage progenitors (GMP). Since the WNT/β-catenin pathway is usually active in HSCs, but not in GMP, these results suggest that the reactivation of β-catenin signal is necessary for some oncogenes to transform progenitor cells (103). In T-ALL, WNT signaling pathway can specifically support LSC and promote tumor growth (104). Studies by Lu et al. have shown that ionic changes caused by salinomycin and related drugs inhibit proximal WNT signaling by interfering with LPR6 phosphorylation, thus impairing the survival of CLL cells dependent on WNT signaling on the plasma membrane (105). Yahata’s team found that the TGF-β/PAI-1 signaling axis was selectively enhanced in the bone marrow of CML-LSC, while PAI-1 enhanced the sensitivity of CML-LSCs to TKI, thus promoting CML-LSCs eradication and leading to sustained remission of the disease (106).

Collectively, since the above signaling pathways are closely related to leukemia, and the former can be regulated by TRAF6. Then TRAF6 is very likely to affect the biological function of leukemia by participating in specific signal pathways. By synthesizing the related studies of TRAF6 in leukemia, we explore the role of TRAF6 in leukemia and try to find evidence that TRAF6 can be a target for leukemia therapy.

#### 4.7.1 TRAF6 in ALL

B precursor ALL is the most common malignant tumor in pediatrics (107). In order to explore the possible causes of heterogeneity of therapeutic effect of pediatric B precursor ALL and to establish potential new therapeutic targets, Weston et al. collected bone marrow mononuclear cells from 40 ALL patients and established DNA damage model after 5Gy ionizing radiation (IR)(cobaltCo60). The results showed that in 40 ALL tumors, 21 cases showed normal DNA damage response after IR, with the accumulation of p53 and p21 proteins and the cleavage of caspase3, 7, 9 and PARP1. The remaining 19 tumors showed apoptotic resistance and are lack of caspase3, 7, 9 and PARP1 cleavage. Among the 19 tumors without normal DNA damage reaction, 15 tumors showed abnormal high expression of TRAF5, TRAF6 and cIAP1 after IR, indicating that apoptotic resistance may be related to the activation of NF- κB signaling pathway. In the follow-up study, a highly active PARP1 mutation was found in one of the tumors, suggesting an increasing activity of NF-κB signaling pathway. Inhibition of PARP1 can promote p53-dependent apoptosis after IR by reducing the DNA binding and transcriptional ability of NF-κB. This study suggests that PARP1 may be the therapeutic target of ALL, and TRAF6 participates in the inhibition of PARP1 on DNA damage response by affecting the activation of NF- κB signaling pathway (81).

MyD88/IRAK signaling pathway is very important for the survival of T cells (108–111). However, the function of this pathway in T cell-ALL (T-ALL) is unknown. Li et al. found that the mRNA, protein and phosphorylated protein levels of IRAK1/4 in T-ALL cells were significantly increased. Inhibition of IRAK1/4 with shRNA or small molecular inhibitor can significantly inhibit the proliferation of patient-derived or mouse leukemia model-derived T-ALL cells. Activating IRAK signal through TLR can promote the progress of T-ALL. However, because IRAK inhibition moderately reduced the expansion of T-ALL *in vivo* and did not significantly affect apoptosis, the researchers identified a combination regimen with strong cytotoxicity by screening 484 FDA-approved drugs. The combination of IRAK1/4 inhibitor with ABT-737 or vincristine significantly reduced the leukemia burden and prolonged the survival time of mice. Mechanism studies have shown that IRAK1/4 can activate TRAF6, TRAF6 interacts with MCL1 and activated TRAF6 can promote the ubiquitination of the K63-linked ubiquitination of MCL1, thus maintaining the stability of MCL1 protein. When inhibiting IRAK1/4, the stability of MCL-1 decreases, which makes T-ALL cells more sensitive to combined therapy. This study confirmed that TRAF6 participates in IRAK1/4 signaling pathway and plays an important role in the development of T-ALL (112).

**Figure 3:**
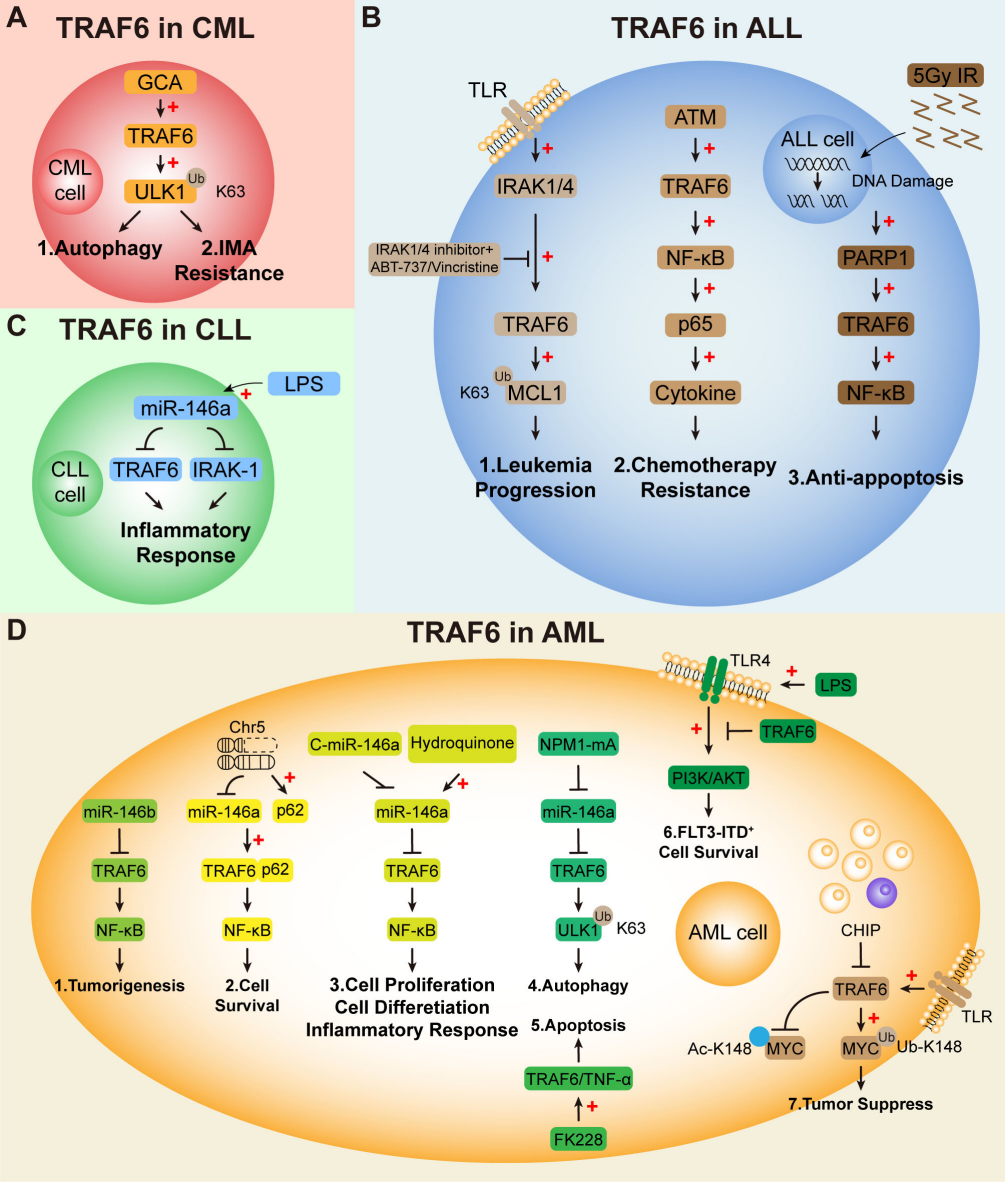
TRAF6 and its related signaling pathways play an important role in leukemia. TRAF6 regulates leukemia pathogenesis through multiple context-dependent mechanisms. **(A)** In CML, GCA promotes TRAF6-mediated ULK1 ubiquitination, driving autophagy and imatinib resistance. **(B)** In ALL, DNA damage activates the ATM-TRAF6-NF-κB pathway, promoting chemoresistance through cytokine expression. Radiation-induced PARP1 reduction enhances TRAF6-NF-κB signaling, decreasing apoptosis. In T-ALL, IRAK1/4 activates TRAF6, which K63-ubiquitinates MCL1, stabilizing this anti-apoptotic protein. **(C)** In CLL, LPS stimulation reduces miR-146a, elevating TRAF6 and IRAK1 to trigger inflammatory responses and increase infection susceptibility. **(D)** In AML, the miR-146a/TRAF6/NF-κB axis is central: miR-146a suppresses TRAF6 expression, while low miR-146a (as in del(5q) AML) or high p62 activates TRAF6-NF-κB signaling, promoting cell survival. NPM1 mutations suppress miR-146a, enhancing TRAF6-mediated K63-ubiquitination of ULK1 to drive autophagy. C-miR-146a conjugates therapeutically target this axis. TRAF6 also exhibits tumor-suppressive functions by antagonizing MYC acetylation in CHIP-associated contexts, while LPS/TLR-4 signaling modulates TRAF6 effects on PI3K/AKT in FLT3-ITD+ cells. HDAC inhibitors (FK228) induce apoptosis via TNF-α/TRAF6 upregulation.

BM niche regulates the dynamic balance of hematopoietic stem cells (HSCs), and also participates in the pathogenesis and drug resistance of ALL. The cross reaction between chemotherapy induced cytokines secreted by ALL cells and BM niche is one of the mechanisms of ALL drug resistance (113, 114). In order to explore the mechanism, Chen et al. conducted relevant research and found that the expression level of niche-protection-related cytokines is increased in all cell lines and primary cells after chemotherapy. ATM dependent DNA damage response pathway mediates the up regulation of these cytokines, and chemotherapy activated NF- κB pathway is also involved in regulating cytokine expression. Further research shows that ATM activates TRAF6 which functions as a signal transducer in the NF-κB pathway through TRAF6 binding motif to activate NF-κB pathway, NF-κB transcription factor p65 directly regulates cytokine expression. Inhibition of ATM or p65 can significantly reduce the residual ALL cells in the xenograft mouse models after Ara-C treatment. This study found a new mechanism of ALL drug resistance involving TRAF6, targeting ATM/TRAF6/NF-κB/p65 signal axis can effectively eradicate chemotherapy resistant ALL cells and increase the sensitivity of ALL to chemotherapy (115) (**Figure 3B**).

#### 4.7.2 TRAF6 in AML

MicroRNAs, a class of important epigenetic regulatory molecules, can regulate the expression of more than 1/3 human genes by inhibiting mRNA translation or degrading genes in the post-transcriptional stage (116). The regulation of microRNAs is involved in many biological processes, including cell proliferation and apoptosis, growth and development, cell metabolism and cell differentiation (117). MiR-146a is a member of the miR-146miRNA family located on chromosome 5. It is a negative feedback regulator of TRAF6 and IRAK1, and the latter two act as downstream adaptor proteins of Toll-like receptors. MiR-146a also participates in the regulation of NF- κ B transcriptional activity and plays an important role in hematopoietic differentiation and cellular immunity (118–121). Existing studies have shown that miR-146a/TRAF6/NF-κB axis plays an important role in AML. (**Figure 3D**).

##### 4.7.2.1 The miR-146a/TRAF6/NF-κB regulatory axis

Deletion of chromosome 5Q (del5q) is common in high-risk AML (HR AML). However, the mechanism of gene regulation involved is not clear. It has been found that decreased expression of miR-146a can activate downstream TRAF6/NF-κB in HR AML and hematopoietic stem/progenitor cells (HSPC) with del (5Q). Del (5Q) HR AML HSPC with low expression of miR-146a showed increased survival and proliferation, and the adjacent haploid gene SQSTM1 (p62), expressed by the intact 5Q allele, could promote this phenomenon. Further studies have shown that the over-expression of the intact allele p62 is due to the feedforward signals from activated NF-κB which is caused by lack of miR-146a. P62 is essential for TRAF6/NF-κB. Inhibition of p62-TRAF6 complex can lead to cell cycle arrest and apoptosis of AML cells. This study shows that del (5Q) HRAML enhances the downstream TRAF6/NF- κ B signal through the loss of miR-146and the overexpression of p62 haploid, thus promoting the maintenance and proliferation of AML cells. Targeting p62/TRAF6/NF-κB can be used as a therapeutic option for del (5Q) HR AML (122).

Yuan et al. studied the key mechanism of regulating benzene-induced hematotoxicity and even leukemia and the expression of miR-146a in the differentiation of human CD34^+^ hematopoietic progenitor cells (HPC) and human acute promyelocytic leukemia cells (HL-60). First, they found that hydroquinone can inhibit erythroid cell colony formation *in vitro*, promote myeloid cell colony formation, and inhibit the proliferation and differentiation of CD34^+^ cells *in vitro*. Then, they carried out related experiments to clarify the mechanism of inhibition of HPCs differentiation induced by hydroquinone. QRT-PCR and western blotting results showed that hydroquinone inhibited the proliferation and differentiation of CD34^+^HPCs, up-regulated the expression of miR-146a and down-regulated the expression of TRAF6 and NF-κB in the early stage of differentiation. Inhibition of miR-146a expression by inhibitor could alleviate the inhibitory effect of hydroquinone on myeloid differentiation and restore the protein level of TRAF6 and phosphorylated IκB α as well as the transcriptional activity of NF-κB. This study explains the mechanism of benzene-induced myeloid differentiation disorder and subsequent leukemia, and proves that miR-146a/TRAF6/NF-κB signal axis plays an important role in the occurrence and development of leukemia (123).

MiR-146b and miR-146a have the same seed sequence, which has been shown to lead to inflammatory diseases and tumors by inhibiting the expression of key molecules needed for NF- κ B activation (124–126). In order to explore the functional and physiological differences between them in the process of disease, Mitsumura et al. obtained miR-146b gene knockout (KO) mice and miR-146a-KO mice by genome editing. The results suggest that both lines develop hematological malignancies such as B-cell lymphoma and AML during their growth. However, there are histological differences in the tumors caused by the two, and the malignant rate of tumors in the miR-146a group is higher. Under mitotic stimulation, the expression of both increased, but the expression of miR-146b was lower than that of miR-146a. Both of them can target the same mRNAs, including TRAF6, and inhibit the subsequent NF-κB activity. In conclusion, this study clarified the functional and physiological differences between miR-146b and miR-146a in the pathogenesis of B-cell lymphoma and AML, and confirmed that miR-146b/TRAF6/NF-κB signal axis can also play an important role in AML (127).

Although NF-κB plays a key regulatory role in leukemia, it is still challenging to target NF-κB with drug inhibitors. According to the above, miR-146a can well inhibit the transcriptional activity of NF-κB, making the synthetic miR-146a mimic an attractive opportunity for immune regulation or elimination of carcinogenic signals. However, the effective delivery of miRNA therapeutic agents is challenging, complicated by safety problems and potential non-target effects (128, 129). Several types of miRNA delivery carriers, including liposomes, lipid nanoparticles, dendrimers or hydrogels, have been previously tested and only a few have entered preliminary clinical trials (130–132). Su et al. described a method of targeted delivery of chemically modified miR-146a mimic into myeloid cells and verified the activity of miR-146a mimic in inflammatory and myeloproliferative disease models. The C-miR146a conjugate is synthesized by connecting the CpG-D19 to the miR-146a passenger chain. Both C-miR-146a and miR-146a can inhibit the expression of IRAK1 and TRAF6 and block the activation of NF-kB in target cells, but the former can be rapidly internalized and transported to the cytoplasm of target myeloid cells and leukemic cells. Intravenous injection of C-miR-146a into miR-146a deficient mice could weaken the activation of NF-κB in myeloid cells, alleviate bone marrow proliferation and bacterial hypersensitivity in mice. C-miR-146a can also significantly inhibit the cytokine release syndrome induced by CD19 chimeric antigen receptor (CAR)-T cells, without affecting its anti-tumor activity. In addition, through the cancer genome map AML dataset, they found that the level of miR-146a was negatively correlated with the level of NF-κB. C-miR-146a can induce cytotoxicity of MDSL, HL-60 and MV4–11 leukemia cells. Repeated intravenous injection of C-miR-146a can down-regulate the expression of NF-κB, and then slow down the progression of HL-60 leukemia. This study confirmed that C-miR-146a targeting miR-146a/TRAF6/NF-κB signal axis is expected to become a new therapeutic strategy for patients with myeloproliferative diseases, especially in patients with AML (133).

##### 4.7.2.2 TRAF6-mediated ubiquitination in AML

AML gene mutation is closely related to the prognosis of patients. Nucleophosmin(NPM1) mutation is the most common genetic change in AML. Previous studies have reported that NPM1 mutants promote autophagy and help leukemia cells survive, but the molecular mechanism is not clear (134). Tang et al. found that ULK1, a key regulator of autophagy, is highly expressed in NPM1-mA-positive OCI-AML3 cells and primary NPM1 mutant AML cells. Western blotting showed that NPM1-mA could interact with ULK1 and positively regulate the protein expression of ULK1. High expression of ULK1 could activate autophagy and promote the proliferation of AML cells. Further studies on the mechanism suggest that NPM1-mA can promote the expression of TRAF6 by inhibiting miR-146a, and then enhance the K63-linked ubiquitination of ULK1 mediated by TRAF6, and finally maintain the stability and activity of ULK1. This study found a new autophagy regulation pathway in AML, which helps us to better understand the biological characteristics of AML with NPM1 mutation (135).

##### 4.7.2.3 TRAF6 in PI3K/AKT signaling

FLT3-ITD (internal tandem duplication) is the most common subtype of FLT3 mutation and is associated with poor prognosis because of the high recurrence rate after routine chemotherapy (136–139). Schnetzke’s team studied the effect of stimulation of TLR-4 with lipopolysaccharide (LPS) on the activation of PI3K/AKT pathway in FLT3-ITD-positive AML cells and the role of TRAF6, which mediates TLR signal transduction in it. First, they found that TLR-4 is widely expressed in AML cells, and LPS can activate TLR-4-related pathways. Then when they knocked down TRAF6 in MV4-11 (with FLT3-ITD) cells, the level of phosphorylated AKT (p-AKT) increased significantly. Under the stimulation of LPS, the increase of p-AKT was higher. At the same time, MV4–11 cells with low TRAF6 were less sensitive to cytarabine or daunorubicin. Finally, their results showed that TRAF6 deletion did not affect the expression of MCL-1 protein, but inhibited the sensitivity to proteasome. This study provides evidence that TRAF6 plays an important role in signal transduction and survival of AML cells. Targeting TRAF6 may be a potential therapeutic strategy for AML patients with FLT3-LTD (140).

##### 4.7.2.4 TRAF6 in TNF signaling

In addition to miR-146a/TRAF6/NF-κB signal axis, TRAF6 also plays a regulatory role in AML through other pathways. Sutheesophon et al. analyzed the role of histone deacetylase (HDAC) inhibitor-FK228 in anti-leukemia in myeloid leukemia cell lines HL-60 and K562. Their results suggest that the expression of TNF-α and molecules involved in TNF signal transduction such as TRAF6 are up-regulated in FK228-treated cells. At the same time, FK228 can activate caspase-8/10, and then cleavage caspase-3/7, resulting in the death of these two kinds of cells. The use of neutralizing antibodies or siRNA to inhibit TNF signaling pathway can inhibit caspase cascade reaction and apoptosis. This study confirmed that HDAC inhibitors can fight leukemia by affecting TNF-α/TRAF6 related pathways (141).

##### 4.7.2.5 TRAF6 as a tumor suppressor in AML

In a healthy state, hematopoietic stem cells (HSCs) acquire somatic mutations over time. Although most of these mutations are insignificant, specific mutations provide HSC with a competitive advantage, leading to clonal hematopoiesis of indeterminant potential (CHIP) (142–144). This kind of CHIP increases the risk of developing myeloid malignant tumors in healthy people. In order to identify the signal state that cooperate with preleukemia cells, Muto et al. carried out RNAi screening experiments *in vivo*, and TRAF6 was identified as one of the most prominent genes. In preleukemic cells, the absence of TRAF6 can lead to myeloid leukemia. By comparing the data, they found that AML patients with low expression of TRAF6 had a shorter survival time. In a considerable number of patients with MPN and AML, the loss or inhibition of TRAF6 can occur through a variety of molecular and genetic mechanisms. Extensive meta-analysis of AML patients with stratified expression of TRAF6 was conducted using BEAT-AML data sets. The results suggest that TRAF6 plays a tumor suppressor role in myeloid tumors. More importantly, they found that TLR-induced TRAF6 activation can inhibit the functional activity of MYC by antagonizing the acetylation of MYC, while TRAF6 loss can activate MYC gene and promote the occurrence of acute leukemia. This study found for the first time that TRAF6 can regulate the activity of MYC. At the same time, this study suggests that TRAF6 plays a tumor inhibitory role in AML, and the absence of TRAF6 will lead to the occurrence of leukemia, which is inconsistent with other studies. Collectively, the complex regulatory network of TRAF6 in AML needs more basic research to supplement (145).

#### 4.7.3 TRAF6 in CLL

For decades, infection has been considered to have a significant impact on CLL patients. Several studies have shown that more than 50% of CLL patients suffer from pathogen colonization, resulting in 70% of patients’ deaths. The causes of infection are diverse, including hypogammaglobulinemia, alkylation drug therapy, prudence and persistent T cell immunodeficiency caused by purine analogs and mAbs (146–148). Endotoxin tolerance (ET) is a complex, coordinated and anti-inflammatory reaction, which can increase the risk of infection (149). In order to evaluate the impact of ET on the infection risk of CLL patients, Jurado-Camino et al. separated monocytes from 70 CLL patients and treated them with LPS for three hours ex vivo to generate ET status. Their results showed that these treated cells showed the characteristics of ET, including low cytokine production (IL-1β, IL-6, IL-10 and TNF- α), high phagocytic activity and impaired antigen presentation. At the same time, compared with the basic state, the expression of miR-146a in monocytes stimulated by LPS increased, while the mRNA levels of TNF-α, IRAK-1 and TRAF6 showed significant down-regulation. The down-regulation of these factors can explain the adverse inflammatory reaction observed after endotoxin challenge. Similar results were obtained *in vitro*. In conclusion, this shows that patients with CLL are locked in a refractory state, and no matter what their Ig level is, they are unable to make a typical response to pathogens. Because of the direct contact between tumor and monocytes, monocytes reprogram their inherent response and express high levels of miR-146a, which may regulate the levels of key factors in inflammatory response, such as IRAK1 and TRAF6. These data are the first to report the cross tolerance between endotoxin and tumor, highlighting the regulatory role of TRAF6 in inflammatory response, and providing a new explanation for the infection risk of CLL patients (150) (**Figure 3C**).

#### 4.7.4 TRAF6 in CML

Imatinib is the first generation TKI targeting BCR-ABL for CML and is still the first-line treatment for patients with CML-CP at present. However, some CML patients are resistant to imatinib, which eventually leads to treatment failure (151–154). Kim and his colleagues studied the molecular mechanism of imatinib resistance in CML. The results suggest that GCA (grancalcin), a cytosolic protein that is translocated to the granule membrane upon neutrophil activation, are highly expressed in imatinib-resistant CML-CP patients, and imatinib resistance induced by GCA is mainly in p-CRKL-positive CML cells. Functional experiments show that GCA can inhibit imatinib-induced apoptosis in K562 cells and primary cells, and act as a positive regulator of autophagy in CML. In terms of mechanism, they proved that GCA can stabilize and activate ULK1, a key regulator of autophagy, by activating the activity of TRAF6 and promoting the K63-linked ubiquitination of ULK1 mediated by TRAF6. Further studies have confirmed that GCA positively regulates ULK1 through TRAF6, induces autophagy in CML cells, and eventually leads to imatinib resistance. This study not only found that GCA can induce autophagy for the first time, but also clarified the new mechanism of CML resistance. Targeting GCA/TRAF6/ULK1 signal axis may become a new therapeutic strategy for TKI-resistant CML patients in the future (155) (**Figure 3A**).

The extensive research reviewed above demonstrates that TRAF family members, particularly TRAF6, participate in complex regulatory networks governing leukemia pathogenesis across diverse disease subtypes. However, translating these mechanistic insights into clinical applications requires critical synthesis of the sometimes-contradictory findings, identification of unifying principles, and assessment of therapeutic opportunities and challenges. In the concluding section, we integrate these findings to provide a comprehensive perspective on TRAF biology in leukemia and discuss the path forward for therapeutic development.

## 5 Therapeutic potential and challenges in targeting TRAFs

### 5.1 Current therapeutic strategies targeting TRAFs

The biological prominence of TRAF family members in leukemogenesis positions them as compelling therapeutic targets. However, translating these findings into clinical practice remains a formidable challenge. Current strategies can be broadly categorized into direct and indirect approaches. Direct targeting primarily focuses on disrupting TRAF-receptor interactions or inhibiting their E3 ubiquitin ligase activity. For instance, the TRAF1/cIAP axis in CLL has been targeted using PKN1 inhibitors, such as OTSSP167 and XL-228 (47). Conversely, indirect strategies circumvent the difficulties of targeting cytosolic adaptor proteins by modulating upstream regulators (e.g., IRAK1/4 inhibitors) or downstream effectors such as the NF-κB and PI3K/AKT pathways (112).Notably, microRNA-based approaches—particularly miR-146a mimics—have emerged as potent epigenetic tools to recalibrate TRAF6 expression in AML and CLL (133).

### 5.2 Challenges in drug development

The scarcity of TRAF-specific inhibitors in clinical trials is largely attributed to several structural and biological hurdles. First, the high structural homology within the TRAF domain across family members complicates the development of highly selective small molecules, posing a significant risk of off-target toxicity. Second, because TRAFs are central scaffolds in innate and adaptive immunity, systemic inhibition may lead to profound immunosuppression or paradoxical inflammatory responses. Furthermore, the functional redundancy observed among TRAFs suggests that leukemia cells may utilize compensatory signaling bypasses, potentially leading to rapid therapeutic resistance.

### 5.3 Safety

A critical caveat in targeting the TRAF family, particularly TRAF6, lies in its biphasic role in myeloid malignancies. As discussed previously, TRAF6 can transition from an oncogene in established AML to a tumor suppressor in specific genetic or developmental contexts (e.g., during the MDS-to-AML progression). This functional implies that inhibition could inadvertently accelerate disease progression in certain patient subsets. Therefore, therapeutic intervention must move beyond simple inhibition toward homeostatic normalization.

### 5.4 Future directions

To overcome the “undruggable” nature of protein-protein interactions, innovative technologies such as Proteolysis-Targeting Chimeras (PROTACs) offer a promising avenue for the selective degradation of specific TRAF members. Moreover, the future of TRAF-targeted therapy is inextricably linked to precision medicine. The integration of longitudinal single-cell sequencing and functional genomics will be essential to identify signatures for biomarker-guided stratification. Combining TRAF modulation with existing regimens (e.g., TKIs in CML or FLT3 inhibitors in AML) may provide the synergistic pressure necessary to eradicate leukemic stem cell populations.

TRAF family members represent biologically compelling therapeutic targets in leukemia, with extensive preclinical evidence supporting their roles in disease pathogenesis, progression, and treatment resistance. However, successful clinical translation will require overcoming significant challenges related to specificity, delivery, context-dependent functions, and safety. Continued investment in basic research to clarify TRAF biology, coupled with innovative drug development and rational clinical trial design, will be essential to realize the therapeutic potential of TRAF-targeted interventions in leukemia.

## 6 Conclusions

**Figure 4:**
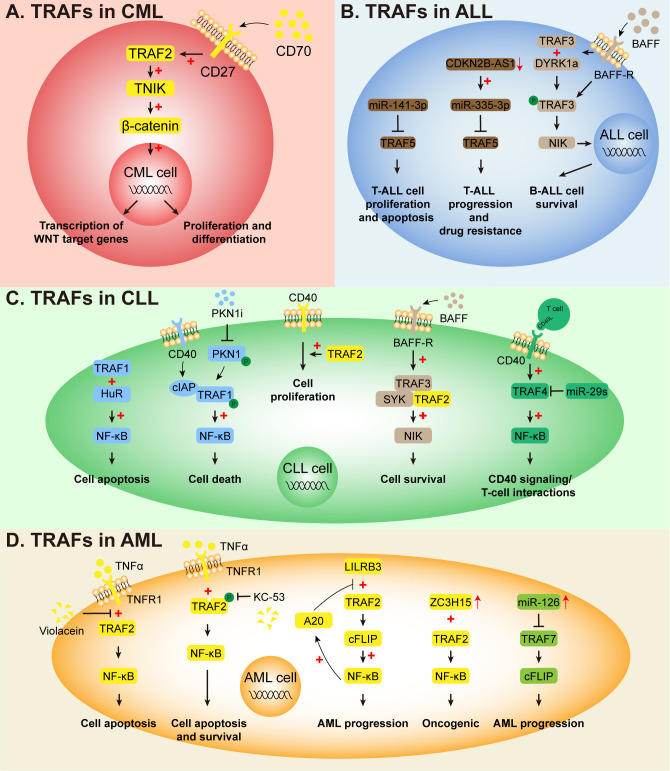
The role of other TRAFs in leukemia. **(A)** In CML, CD27-CD70 ligation activates TRAF2 and β-catenin, driving WNT target gene expression and LSC self-renewal. **(B)** In ALL, DYRK1a phosphorylates TRAF3 upon BAFF stimulation, interfering with NIK degradation and activating non-canonical NF-κB in B-ALL. MiR-141-3p and miR-335-3p target TRAF5, regulating T-ALL cell survival, apoptosis, and chemosensitivity. **(C)** In CLL, multiple TRAF-dependent pathways regulate disease biology. PKN1 protects TRAF1 from cIAP-mediated degradation during CD40 signaling; PKN1 inhibition (OTSSP167, XL-228) destabilizes TRAF1, inducing cell death. HuR stabilizes TRAF1; HuR inhibition downregulates TRAF1, enhancing apoptosis and drug sensitivity. TRAF2 is essential for CD40-mediated proliferation; TRAF2 deletion paradoxically promotes CLL by rendering cells BAFF-independent. BAFF-R engagement promotes SYK interaction with TRAF2/3 complex, mediating survival signals. MiR-29s suppress TRAF4, impairing CD40-NF-κB activation; BCR inhibitors restore miR-29s to block TRAF4 upregulation. **(D)** In AML, TRAF2 activates NF-κB downstream of TNFR1; violacein disrupts TRAF2-TNFR1 interaction to induce apoptosis, while KC-53 inhibits TNF-α-induced TRAF2 phosphorylation, blocking p65 nuclear translocation. LILRB3 recruits cFLIP via TRAF2, activating NF-κB and suppressing anti-tumor T cells. ZC3H15 interacts with TRAF2, potentially mediating NF-κB signaling. TRAF7 functions as a tumor suppressor; miR-126 overexpression downregulates TRAF7, blocking c-FLIP and promoting leukemogenesis.

Taken together, seven members of the TRAF family perform different functions in leukemia. (**Table 1**) Their biological characteristics are similar, but they are also different. First of all, in view of the characteristics of the TRAF family described above, most members cannot function without the NF-κB signaling pathway. Many drugs, small molecule inhibitors or genes interfere with the expression of TRAFs by affecting the NF-κB signal pathway, and then regulate the activity of leukemic cells. (**Table 2**) Secondly, the role of TRAF family members in leukemia seems to be slightly different. Collected studies have confirmed that TRAF1, TRAF3 and TRAF4 play the role of oncogenes in leukemia, TRAF7 has anti-leukemia activity, while the role of TRAF2 and TRAF6 is contradictory. TRAF2 promotes the occurrence and development of leukemia in AML and CML, while studies in CLL have confirmed that the absence of TRAF2 is beneficial to the development of CLL in mice, in view of the numerous studies on TRAF6. We will discuss it in detail later. Thirdly, Leukemias are diverse and heterogeneous. There are similarities and differences in the function and mechanism of TRAFs in different types of leukemia. In AML, TRAF2 and TRAF6 affect the growth, proliferation and apoptosis of leukemia cells mainly by regulating the NF-κB signaling pathway. In addition, TRAF6 can also affect the survival of leukemia cells by mediating the ubiquitination degradation of substrates and regulating the PI3K/AKT signaling pathway. (**Figure 4D**) In CML, TRAF2 acts by regulating the WNT signaling pathway rather than the NF-κB signaling pathway, while TRAF6 influences autophagy and drug resistance of CML cells by mediating ubiquitination degradation of ULK1. (**Figure 4A**) In ALL, TRAF6 still affects the occurrence and development of leukemia by targeting substrate ubiquitination degradation and regulating NF-κB signaling pathway. TRAF5 and TRAF3 can promote the survival of ALL cells, but the downstream mechanism is still unknown. (**Figure 4B**) In CLL, TRAF1 and TRAF4 function through the NF-κB signaling pathway. TRAF2 affects the survival of leukemia cells by activating downstream gene expression of WNT signaling pathway in a mechanism similar to that of CML. (**Figure 4C**) Finally, although we mentioned earlier that TRAFs has two major functions (linker protein and E3 ubiquitin ligase), existing studies have shown that TRAFs mainly plays the role of linker protein in leukemia. In general, TRAFs affects the development of leukemia by regulating NF-κB, PI3K/AKT, and WNT signaling pathways, and a small number of studies have confirmed that TRAFs also plays a further role in leukemia by mediating the ubiquitination degradation of substrates (ULK1, MCL1, p65, MYC).

**Table 1:** The regulatory axis and function of TRAF family members in leukemia.

TRAFs	Leukemia	The regulatory axis	The function
TRAF1	CLL	PKN1/TRAF1	Cell death
TRAF1	CLL	HuR/TRAF1	Cell proliferation and apoptosis
TRAF2	AML	ZC3H15/TRAF2/NF-κB	Leukemia development
TRAF2	AML	LILRB3/TRAF2	Leukemia progression
TRAF2	CLL	CD40/TRAF2/p38	Leukemia development
TRAF2	CML	(CD27+CD70)/TRAF2/WNT	LSCs proliferation and growth
TRAF2	HAP1 cell	TRAF2/JNK	Autophagy
TRAF2+TRAF3	CLL	SYK/(TRAF2+TRAF3)/BAFF	Cell apoptosis
TRAF3	B-ALL	DYRK1a/TRAF3/NIK	Leukemia development
TRAF4	CLL	miR-29s/TRAF4/CD40	Leukemia development
TRAF5	T-ALL	CDKN2B-AS1/miR-335-3p/TRAF5	Leukemia development and drug resistance
TRAF5	T-ALL	MiR-141-3p/TRAF5	Cell apoptosis
TRAF6	CML	GCA/TRAF6/ULK1	Autophagy and IMA resistance
TRAF6	CLL	miR-146a/TRAF6	Inflammatory response
TRAF6	ALL	(IRAK1/4)/TRAF6/MCL1	Leukemia progression
TRAF6	ALL	ATM/TRAF6/NF-κB	Chemotherapy resistance and apoptosis
TRAF6	AML	TRAF6/NF-κB	Tumorigenesis, cell survival, cell proliferation, cell differentiation and inflammatory response
TRAF6	AML	miR-146a/TRAF6/ULK1	Autophagy
TRAF6	AML	TRAF6/PI3K/AKT	Cell apoptosis
TRAF6	AML	TRAF6/MYC	Tumorigenesis, cell survival, cell proliferation and cell differentiation
TRAF7	Myeloid cells	TRAF7/KLF2/PFKFB3	Cell cycle and leukemia development

**Table 2:** Potential drugs, compounds, natural extracts and small molecule inhibitors targeting TRAFs that can be used in leukemia.

Name	Type	Leukemia	Targeting TRAFs
OTSSP167/XC-228	Small molecule inhibitors	CLL	TRAF1
Indole-3-carbinol	Natural extracts	Myeloid and leukemic cells	TRAF1
CDDO-Me	Synthetic compound	Leukemic cells	TRAF1
Gambogic acid	Natural extracts	Leukemic cells	TRAF1
Violacein	Natural extracts	AML	TRAF2
KC-53	Natural extracts	AML	TRAF2
miR-29s	microRNA	CLL	TRAF4
miR-335-3p	microRNA	T-ALL	TRAF5
miR-141-3p	microRNA	T-ALL	TRAF5
miR-146a	microRNA	CLL/AML	TRAF6
miR-146b	microRNA	AML	TRAF6
IRAK1/4 inhibitor	Small molecule inhibitors	ALL	TRAF6
Hydroquinone	Natural extracts	AML	TRAF6
FK228	Small molecule inhibitors	AML	TRAF6

In general, the function and role of TRAF6 in leukemia was not significantly different from that in other tumors. Based on the existing studies of TRAF6 in leukemia, TRAF6 is overexpressed in ALL, CLL and CML, which plays a tumor-promoting effect and has a good chance to become a therapeutic target. But in AML, the conclusions seem to be contradictory. Although most of these studies believe that targeting overexpressed TRAF6 can fight AML, in a study in 2022, the authors found that TRAF6 is a tumor suppressor, and the loss of TRAF6 can promote the occurrence of acute leukemia. TRAF6 is the regulator of HSC homeostasis and it has been reported that the expression of TRAF6 in MDS HSPCs is widely increased (156, 157). In this case, TRAF6 overexpression leads to MDS-associated HSPC defects. The paradoxical observation that TRAF6 low expressing AML is overexpressed in MDS but reduced in expression and function in a subset of MPN/AML patients may indicate that TRAF6^low^ AML emerges from a different subclone rather than developing progressively from MDS. Taken together, these findings reveal that cell states are sensitive to TRAF6 dose and TRAF6 exhibits both proto-oncogenic and tumor suppressor functions. More evidence is needed to determine which is correct. In leukemia, TRAF6 is an important signal pathway regulator, which fully plays the role of the adaptor protein and E3 ubiquitin ligase. Among the regulatory pathways involved in TRAF6, NF-κB signaling pathway is the most common, followed by PI3K/AKT pathway. It is rich in upstream proteins, including PARP1, IRAK1/4, ATM, TNF-α, NPM1-mA, GCA and TLR-4. As an E3 ubiquitin ligase, TRAF6 mediates the K63-linked ubiquitination of downstream substrates, including MCL1 and ULK1, activating and maintaining their stability. In addition, in AML, TRAF6 can antagonize the acetylation of MYC to inhibit MYC activity, and also promote the release of IL-8 to activate the inflammatory response. TRAF6’s rich signal regulatory network makes it involved in a variety of biological activities related to leukemia, including cell growth and proliferation, cell differentiation, apoptosis, autophagy, drug sensitivity, inflammation and patient infection.

The conflicting functional of TRAF6 in AML represents a pivotal area for critical evaluation. While the prevailing consensus emphasizes an oncogenic role, where TRAF6-mediated NF-κB/PI3K/AKT activation and substrate ubiquitination (e.g., MCL1, ULK1) drive cell survival and autophagy, recent evidence challenging this paradigm cannot be overlooked. This characteristic likely reflects a complex interplay of context-dependent mechanisms. First, the role of TRAF6 appears stage-specific. The observation that TRAF6 is frequently overexpressed in MDS and certain AML subtypes, yet downregulated in others, suggests a functional evolution during leukemogenesis. Specifically, high TRAF6 levels may drive the initial MDS-associated hematopoietic defects, whereas a subsequent reduction in TRAF6 function might be required to cooperate with specific genetic alterations to facilitate the transition to overt AML. Second, the genetic landscape fundamentally dictates TRAF6 output; for instance, TRAF6 may function oncogenically in the presence of NPM1 or FLT3-ITD mutations, but exert tumor-suppressive effects by antagonizing MYC activity in clones arising from clonal hematopoiesis. Furthermore, a dose-dependent model should be considered, where normal hematopoiesis requires a suitable level of TRAF6 activity—deviations in either direction (excessive signaling or functional deficiency) may trigger distinct oncogenic programs. Finally, Leukemia comprises heterogeneous populations including stem cells, progenitors, and differentiated blasts. TRAF6 functions may differ across these subpopulations, with aggregated analyses obscuring cell-type-specific effects. The distinction between transient knockdown and germline knockout models, or the use of cell lines versus primary patient samples, may further contribute to these divergent observations.

In view of the cancer-promoting characteristics of TRAF6 in leukemia, the development of drugs or small molecular inhibitors targeting TRAF6 directly or indirectly seems to have the opportunity to change the treatment strategy of leukemia. In the existing research, miR-146a is the most studied, directly inhibiting the microRNA of TRAF6 and can affect a variety of biological activities of AML and CLL by targeting inhibition of TRAF6. However, the current number of related studies is limited, and lack of clinical trial data support. In addition, some drugs, small molecular inhibitors or synthetic substances can also exert antileukemic effects by affecting the expression of other TRAF family members, but more data is needed to verify their effects.

In any case, TRAF family members’ existing research on leukemia has outlined the basic molecular regulatory network, and more basic research in the future will greatly enrich the function and role of TRAFs in leukemia. We believe that TRAF family members have great potential to become a target for leukemia, and the development of new drug therapy targeting TRAFs alone or in combination with traditional therapy will bring new dawn to leukemia patients.

## References

[1] Aitken MJL Ravandi F Patel KP Short NJ . Prognostic and therapeutic implications of measurable residual disease in acute myeloid leukemia. J Hematol Oncol. (2021) 14:137. doi: 10.1186/s13045-021-01148-5, PMID: 34479626 PMC8417965

[2] Chan O Renneville A Padron E . Chronic myelomonocytic leukemia diagnosis and management. Leukemia. (2021) 35:1552–62. doi: 10.1038/s41375-021-01207-3, PMID: 33714974

[3] Kantarjian H Kadia T DiNardo C Daver N Borthakur G Jabbour E . Acute myeloid leukemia: current progress and future directions. Blood Cancer J. (2021) 11:41. doi: 10.1038/s41408-021-00425-3, PMID: 33619261 PMC7900255

[4] Hallek M Al-Sawaf O . Chronic lymphocytic leukemia: 2022 update on diagnostic and therapeutic procedures. Am J hematol. (2021) 96:1679–705. doi: 10.1002/ajh.26367, PMID: 34625994

[5] Nguyen HTK Terao MA Green DM Pui CH Inaba H . Testicular involvement of acute lymphoblastic leukemia in children and adolescents: Diagnosis, biology, and management. Cancer. (2021) 127:3067–81. doi: 10.1002/cncr.33609, PMID: 34031876 PMC9677247

[6] Lim SH Dubielecka PM Raghunathan VM . Molecular targeting in acute myeloid leukemia. J Transl Med. (2017) 15:183. doi: 10.1186/s12967-017-1281-x, PMID: 28851395 PMC5576374

[7] Patel K Pagel JM . Current and future treatment strategies in chronic lymphocytic leukemia. J Hematol Oncol. (2021) 14:69. doi: 10.1186/s13045-021-01054-w, PMID: 33902665 PMC8074228

[8] Lew TE Tam CS Seymour JF . How I treat chronic lymphocytic leukemia after venetoclax. Blood. (2021) 138:361–9. doi: 10.1182/blood.2020008502, PMID: 33876212

[9] Isidori A Cerchione C Daver N DiNardo C Garcia-Manero G Konopleva M . Immunotherapy in acute myeloid leukemia: where we stand. Front Oncol. (2021) 11:656218. doi: 10.3389/fonc.2021.656218, PMID: 34041025 PMC8143531

[10] Loke J Buka R Craddock C . Allogeneic stem cell transplantation for acute myeloid leukemia: Who, When, and How? Front Immunol. (2021) 12:659595. doi: doi:10.3389/fimmu.2021.659595, PMID: 34012445 PMC8126705

[11] Dal-Bo M Bertoni F Forconi F Zucchetto A Bomben R Marasca R . Intrinsic and extrinsic factors influencing the clinical course of B-cell chronic lymphocytic leukemia: prognostic markers with pathogenetic relevance. J Transl Med. (2009) 7:76. doi: 10.1186/1479-5876-7-76, PMID: 19715592 PMC2747913

[12] Weber S Parmon A Kurrle N Schnütgen F Serve H . The clinical significance of iron overload and iron metabolism in myelodysplastic syndrome and acute myeloid leukemia. Front Immunol. (2020) 11:627662. doi: 10.3389/fimmu.2020.627662, PMID: 33679722 PMC7933218

[13] Singh V Uddin MH Zonder JA Azmi AS Balasubramanian SK . Circular RNAs in acute myeloid leukemia. Mol cancer. (2021) 20:149. doi: 10.1186/s12943-021-01446-z, PMID: 34794438 PMC8600814

[14] Stevenson FK Forconi F Kipps TJ . Exploring the pathways to chronic lymphocytic leukemia. Blood. (2021) 138:827–35. doi: 10.1182/blood.2020010029, PMID: 34075408 PMC8432043

[15] Marleau AM Lipton JH Riordan NH Ichim TE . Therapeutic use of Aldara in chronic myeloid leukemia. J Transl Med. (2007) 5:4. doi: 10.1186/1479-5876-5-4, PMID: 17254347 PMC1790884

[16] Gupta I Varshney NK Khan S . Emergence of members of TRAF and DUB of ubiquitin proteasome system in the regulation of hypertrophic cardiomyopathy. Front Genet. (2018) 9:336. doi: 10.3389/fgene.2018.00336, PMID: 30186311 PMC6110912

[17] Xie P Kraus ZJ Stunz LL Bishop GA . Roles of TRAF molecules in B lymphocyte function. Cytokine Growth factor Rev. (2008) 19:199–207. doi: 10.1016/j.cytogfr.2008.04.002, PMID: 18499506 PMC2495079

[18] Wang Y Zhang P Liu Y Cheng G . TRAF-mediated regulation of immune and inflammatory responses. Sci China Life Sci. (2010) 53:159–68. doi: 10.1007/s11427-010-0050-3, PMID: 20596822

[19] Wu L Chen X Zhao J Martin B Zepp JA Ko JS . A novel IL-17 signaling pathway controlling keratinocyte proliferation and tumorigenesis via the TRAF4-ERK5 axis. J Exp Med. (2015) 212:1571–87. doi: 10.1084/jem.20150204, PMID: 26347473 PMC4577838

[20] Wang Q Gao G Zhang T Yao K Chen H Park MH . TRAF1 is critical for regulating the BRAF/MEK/ERK pathway in non-small cell lung carcinogenesis. Cancer Res. (2018) 78:3982–94. doi: 10.1158/0008-5472.CAN-18-0429, PMID: 29748372 PMC6050072

[21] Liang Z Li X Liu S Li C Wang X Xing J . MiR-141-3p inhibits cell proliferation, migration and invasion by targeting TRAF5 in colorectal cancer. Biochem Biophys Res Commun. (2019) 514:699–705. doi: 10.1016/j.bbrc.2019.05.002, PMID: 31078266

[22] Peramuhendige P Marino S Bishop RT de Ridder D Khogeer A Baldini I . TRAF2 in osteotropic breast cancer cells enhances skeletal tumour growth and promotes osteolysis. Sci Rep. (2018) 8:39. doi: 10.1038/s41598-017-18327-5, PMID: 29311633 PMC5758572

[23] Pineda G Ea CK Chen ZJ . Ubiquitination and TRAF signaling. Adv Exp Med Biol. (2007) 597:80–92. doi: 10.1007/978-0-387-70630-6_7, PMID: 17633019

[24] Hacker H Tseng PH Karin M . Expanding TRAF function: TRAF3 as a tri-faced immune regulator. Nat Rev Immunol. (2011) 11:457–68. doi: 10.1038/nri2998, PMID: 21660053

[25] Ha H Han D Choi Y . TRAF-mediated TNFR-family signaling. Curr Protoc Immunol. (2009) Chapter 11:Unit11.9D. doi: 10.1002/0471142735.im1109ds87, PMID: 19918944

[26] Rothe M Wong SC Henzel WJ Goeddel DV . A novel family of putative signal transducers associated with the cytoplasmic domain of the 75 kDa tumor necrosis factor receptor. Cell. (1994) 78:681–92. doi: 10.1016/0092-8674(94)90532-0, PMID: 8069916

[27] Hsu H Xiong J Goeddel DV . The TNF receptor 1-associated protein TRADD signals cell death and NF-kappa B activation. Cell. (1995) 81:495–504. doi: 10.1016/0092-8674(95)90070-5, PMID: 7758105

[28] Wesche H Henzel WJ Shillinglaw W Li S Cao Z . MyD88: an adapter that recruits IRAK to the IL-1 receptor complex. Immunity. (1997) 7:837–47. doi: 10.1016/S1074-7613(00)80402-1, PMID: 9430229

[29] Rothe M Pan MG Henzel WJ Ayres TM Goeddel DV . The TNFR2-TRAF signaling complex contains two novel proteins related to baculoviral inhibitor of apoptosis proteins. Cell. (1995) 83:1243–52. doi: 10.1016/0092-8674(95)90149-3, PMID: 8548810

[30] Zheng C Kabaleeswaran V Wang Y Cheng G Wu H . Crystal structures of the TRAF2: cIAP2 and the TRAF1: TRAF2: cIAP2 complexes: affinity, specificity, and regulation. Mol Cell. (2010) 38:101–13. doi: 10.1016/j.molcel.2010.03.009, PMID: 20385093 PMC2855162

[31] Mace PD Smits C Vaux DL Silke J Day CL . Asymmetric recruitment of cIAPs by TRAF2. J Mol Biol. (2010) 400:8–15. doi: 10.1016/j.jmb.2010.04.055, PMID: 20447407

[32] Vince JE Pantaki D Feltham R Mace PD Cordier SM Schmukle AC . TRAF2 must bind to cellular inhibitors of apoptosis for tumor necrosis factor (tnf) to efficiently activate nf-{kappa}b and to prevent tnf-induced apoptosis. J Biol Chem. (2009) 284:35906–15. doi: 10.1074/jbc.M109.072256, PMID: 19815541 PMC2791019

[33] Sanjo H Zajonc DM Braden R Norris PS Ware CF . Allosteric regulation of the ubiquitin:NIK and ubiquitin:TRAF3 E3 ligases by the lymphotoxin-beta receptor. J Biol Chem. (2010) 285:17148–55. doi: 10.1074/jbc.M110.105874, PMID: 20348096 PMC2878066

[34] Bhoj VG Chen ZJ . Ubiquitylation in innate and adaptive immunity. Nature. (2009) 458:430–7. doi: 10.1038/nature07959, PMID: 19325622

[35] Kashiwada M Shirakata Y Inoue JI Nakano H Okazaki K Okumura K . Tumor necrosis factor receptor-associated factor 6 (TRAF6) stimulates extracellular signal-regulated kinase (ERK) activity in CD40 signaling along a ras-independent pathway. J Exp Med. (1998) 187:237–44. doi: 10.1084/jem.187.2.237, PMID: 9432981 PMC2212104

[36] Rothe M Sarma V Dixit VM Goeddel DV . TRAF2-mediated activation of NF-kappa B by TNF receptor 2 and CD40. Sci (New York NY). (1995) 269:1424–7. doi: 10.1126/science.7544915, PMID: 7544915

[37] Cao Z Xiong J Takeuchi M Kurama T Goeddel DV . TRAF6 is a signal transducer for interleukin-1. Nature. (1996) 383:443–6. doi: 10.1038/383443a0, PMID: 8837778

[38] Häcker H Vabulas RM Takeuchi O Hoshino K Akira S Wagner H . Immune cell activation by bacterial CpG-DNA through myeloid differentiation marker 88 and tumor necrosis factor receptor-associated factor (TRAF)6. J Exp Med. (2000) 192:595–600. doi: 10.1084/jem.192.4.595, PMID: 10952730 PMC2193231

[39] Xu M Skaug B Zeng W Chen ZJ . A ubiquitin replacement strategy in human cells reveals distinct mechanisms of IKK activation by TNFalpha and IL-1beta. Mol Cell. (2009) 36:302–14. doi: 10.1016/j.molcel.2009.10.002, PMID: 19854138 PMC2779160

[40] Zeng W Xu M Liu S Sun L Chen ZJ . Key role of Ubc5 and lysine-63 polyubiquitination in viral activation of IRF3. Mol Cell. (2009) 36:315–25. doi: 10.1016/j.molcel.2009.09.037, PMID: 19854139 PMC2779157

[41] Yamazaki K Gohda J Kanayama A Miyamoto Y Sakurai H Yamamoto M . Two mechanistically and temporally distinct NF-kappaB activation pathways in IL-1 signaling. Sci Signaling. (2009) 2:ra66. doi: 10.1126/scisignal.2000387, PMID: 19843958

[42] Kelliher MA Grimm S Ishida Y Kuo F Stanger BZ Leder P . The death domain kinase RIP mediates the TNF-induced NF-kappaB signal. Immunity. (1998) 8:297–303. doi: 10.1016/S1074-7613(00)80535-X, PMID: 9529147

[43] Alvarez SE Harikumar KB Hait NC Allegood J Strub GM Kim EY . Sphingosine-1-phosphate is a missing cofactor for the E3 ubiquitin ligase TRAF2. Nature. (2010) 465:1084–8. doi: 10.1038/nature09128, PMID: 20577214 PMC2946785

[44] Wong WW Gentle IE Nachbur U Anderton H Vaux DL Silke J . RIPK1 is not essential for TNFR1-induced activation of NF-kappaB. Cell Death differentiation. (2010) 17:482–7. doi: 10.1038/cdd.2009.178, PMID: 19927158

[45] Matsuzawa A Tseng PH Vallabhapurapu S Luo JL Zhang W Wang H . Essential cytoplasmic translocation of a cytokine receptor-assembled signaling complex. Sci (New York NY). (2008) 321:663–8. doi: 10.1126/science.1157340, PMID: 18635759 PMC2669719

[46] Vallabhapurapu S Matsuzawa A Zhang W Tseng PH Keats JJ Wang H . Nonredundant and complementary functions of TRAF2 and TRAF3 in a ubiquitination cascade that activates NIK-dependent alternative NF-kappaB signaling. Nat Immunol. (2008) 9:1364–70. doi: 10.1038/ni.1678, PMID: 18997792 PMC2671996

[47] Edilova MI Law JC Zangiabadi S Ting K Mbanwi AN Arruda A . The PKN1- TRAF1 signaling axis as a potential new target for chronic lymphocytic leukemia. Oncoimmunology. (2021) 10:1943234. doi: 10.1080/2162402X.2021.1943234, PMID: 34589290 PMC8475556

[48] Xiao K Yang L Gao X An Y Xie W Jingquan G . HuR affects proliferation and apoptosis of chronic lymphocytic leukemia cells via NF-kappaB pathway. BioMed Res Int. (2020) 2020:1481572. doi: 10.1155/2020/1481572, PMID: 32908868 PMC7474742

[49] Wang CY Mayo MW Korneluk RG Goeddel DV Baldwin AS Jr . NF-kappaB antiapoptosis: induction of TRAF1 and TRAF2 and c-IAP1 and c-IAP2 to suppress caspase-8 activation. Sci (New York NY). (1998) 281:1680–3. doi: 10.1126/science.281.5383.1680, PMID: 9733516

[50] Takada Y Andreeff M Aggarwal BB . Indole-3-carbinol suppresses NF-kappaB and IkappaBalpha kinase activation, causing inhibition of expression of NF-kappaB-regulated antiapoptotic and metastatic gene products and enhancement of apoptosis in myeloid and leukemia cells. Blood. (2005) 106:641–9. doi: 10.1182/blood-2004-12-4589, PMID: 15811958 PMC1895177

[51] Shishodia S Sethi G Konopleva M Andreeff M Aggarwal BB . A synthetic triterpenoid, CDDO-Me, inhibits IkappaBalpha kinase and enhances apoptosis induced by TNF and chemotherapeutic agents through down-regulation of expression of nuclear factor kappaB-regulated gene products in human leukemic cells. Clin Cancer Res. (2006) 12:1828–38. doi: 10.1158/1078-0432.CCR-05-2044, PMID: 16551868

[52] Pandey MK Sung B Ahn KS Kunnumakkara AB Chaturvedi MM Aggarwal BB . Gambogic acid, a novel ligand for transferrin receptor, potentiates TNF-induced apoptosis through modulation of the nuclear factor-kappaB signaling pathway. Blood. (2007) 110:3517–25. doi: 10.1182/blood-2007-03-079616, PMID: 17673602 PMC2077305

[53] Borghi A Verstrepen L Beyaert R . TRAF2 multitasking in TNF receptor-induced signaling to NF-κB, MAP kinases and cell death. Biochem Pharmacol. (2016) 116:1–10. doi: 10.1016/j.bcp.2016.03.009, PMID: 26993379

[54] Bradley JR Pober JS . Tumor necrosis factor receptor-associated factors (TRAFs). Oncogene. (2001) 20:6482–91. doi: 10.1038/sj.onc.1204788, PMID: 11607847

[55] Ferreira CV Bos CL Versteeg HH Justo GZ Duran N Peppelenbosch MP . Molecular mechanism of violacein-mediated human leukemia cell death. Blood. (2004) 104:1459–64. doi: 10.1182/blood-2004-02-0594, PMID: 15130948

[56] Savva CG Totokotsopoulos S Nicolaou KC Neophytou CM Constantinou AI . Selective activation of TNFR1 and NF-kappaB inhibition by a novel biyouyanagin analogue promotes apoptosis in acute leukemia cells. BMC Cancer. (2016) 16:279. doi: 10.1186/s12885-016-2310-5, PMID: 27098354 PMC4839067

[57] Strausberg RL Feingold EA Grouse LH Derge JG Klausner RD Collins FS . Generation and initial analysis of more than 15,000 full-length human and mouse cDNA sequences. Proc Natl Acad Sci U States A. (2002) 99:16899–903. doi: 10.1073/pnas.242603899, PMID: 12477932 PMC139241

[58] Capalbo G Mueller-Kuller T Koschmieder S Klein HU Ottmann OG Hoelzer D . Characterization of ZC3H15 as a potential TRAF-2-interacting protein implicated in the NFkappaB pathway and overexpressed in AML. Int J Oncol. (2013) 43:246–54. doi: 10.3892/ijo.2013.1924, PMID: 23624947

[59] Deng M Gui X Kim J Xie L Chen W Li Z . LILRB4 signalling in leukaemia cells mediates T cell suppression and tumour infiltration. Nature. (2018) 562:605–9. doi: 10.1038/s41586-018-0615-z, PMID: 30333625 PMC6296374

[60] Gui X Deng M Song H Chen Y Xie J Li Z . Disrupting LILRB4/APOE interaction by an efficacious humanized antibody reverses T-cell suppression and blocks AML development. Cancer Immunol Res. (2019) 7:1244–57. doi: 10.1158/2326-6066.CIR-19-0036, PMID: 31213474 PMC6677629

[61] Anami Y Deng M Gui X Yamaguchi A Yamazaki CM Zhang N . LILRB4-targeting antibody-drug conjugates for the treatment of acute myeloid leukemia. Mol Cancer Ther. (2020) 19:2330–9. doi: 10.1158/1535-7163.MCT-20-0407, PMID: 32879051 PMC7921214

[62] Wu G Xu Y Schultz RD Chen H Xie J Deng M . LILRB3 supports acute myeloid leukemia development and regulates T-cell antitumor immune responses through the TRAF2-cFLIP-NF-κB signaling axis. Nat cancer. (2021) 2:1170–84. doi: 10.1038/s43018-021-00262-0, PMID: 35122056 PMC8809885

[63] Perez-Chacon G Llobet D Pardo C Pindado J Choi Y Reed JC . TNFR-associated factor 2 deficiency in B lymphocytes predisposes to chronic lymphocytic leukemia/small lymphocytic lymphoma in mice. J Immunol. (2012) 189:1053–61. doi: 10.4049/jimmunol.1200814, PMID: 22711886 PMC3526975

[64] Perez-Chacon G Zapata JM . The Traf2DNxBCL2-tg mouse model of chronic lymphocytic leukemia/small lymphocytic lymphoma recapitulates the biased IGHV gene usage, stereotypy, and antigen-specific HCDR3 selection of its human counterpart. Front Immunol. (2021) 12:627602. doi: 10.3389/fimmu.2021.627602, PMID: 33912159 PMC8072112

[65] Zapata JM Krajewska M Morse HC 3rd Choi Y Reed JC . TNF receptor-associated factor (TRAF) domain and Bcl-2 cooperate to induce small B cell lymphoma/chronic lymphocytic leukemia in transgenic mice. Proc Natl Acad Sci U States A. (2004) 101:16600–5. doi: 10.1073/pnas.0407541101, PMID: 15545599 PMC534512

[66] Schurch C Riether C Matter MS Tzankov A Ochsenbein AF . CD27 signaling on chronic myelogenous leukemia stem cells activates Wnt target genes and promotes disease progression. J Clin Invest. (2012) 122:624–38. doi: 10.1172/JCI45977, PMID: 22232214 PMC3266773

[67] Palumbo C Mecchia A Bocedi A Aquilano K Lettieri-Barbato D Rosina M . Revisited role of TRAF2 and TRAF2 C-terminal domain in endoplasmic reticulum stress-induced autophagy in HAP1 leukemia cells. Int J Biochem Cell Biol. (2022) 145:106193. doi: 10.1016/j.biocel.2022.106193, PMID: 35257890

[68] Perez-Carretero C Hernandez-Sanchez M Gonzalez T Quijada-Alamo M Martin-Izquierdo M Santos-Minguez S . TRAF3 alterations are frequent in del-3’IGH chronic lymphocytic leukemia patients and define a specific subgroup with adverse clinical features. Am J hematol. (2022) 97:903–14. doi: 10.1002/ajh.26578, PMID: 35472012

[69] Paiva C Rowland TA Sreekantham B Godbersen C Best SR Kaur P . SYK inhibition thwarts the BAFF - B-cell receptor crosstalk and thereby antagonizes Mcl-1 in chronic lymphocytic leukemia. Haematologica. (2017) 102:1890–900. doi: 10.3324/haematol.2017.170571, PMID: 28838991 PMC5664393

[70] Li Y Xie X Jie Z Zhu L Yang JY Ko CJ . DYRK1a mediates BAFF-induced noncanonical NF-κB activation to promote autoimmunity and B-cell leukemogenesis. Blood. (2021) 138:2360–71. doi: 10.1182/blood.2021011247, PMID: 34255829 PMC8832461

[71] Régnier CH Tomasetto C Moog-Lutz C Chenard MP Wendling C Basset P . Presence of a new conserved domain in CART1, a novel member of the tumor necrosis factor receptor-associated protein family, which is expressed in breast carcinoma. J Biol Chem. (1995) 270:25715–21. doi: 10.1074/jbc.270.43.25715, PMID: 7592751

[72] Li W Peng C Lee MH Lim D Zhu F Fu Y . TRAF4 is a critical molecule for Akt activation in lung cancer. Cancer Res. (2013) 73:6938–50. doi: 10.1158/0008-5472.CAN-13-0913, PMID: 24154876 PMC3856436

[73] Ruan X Zhang R Li R Zhu H Wang Z Wang C . The research progress in physiological and pathological functions of TRAF4. Front Oncol. (2022) 12:842072. doi: 10.3389/fonc.2022.842072, PMID: 35242717 PMC8885719

[74] Seda V Mraz M . B-cell receptor signalling and its crosstalk with other pathways in normal and Malignant cells. Eur J haematol. (2015) 94:193–205. doi: 10.1111/ejh.12427, PMID: 25080849

[75] Sharma S Pavlasova GM Seda V Cerna KA Vojackova E Filip D . miR-29 modulates CD40 signaling in chronic lymphocytic leukemia by targeting TRAF4: an axis affected by BCR inhibitors. Blood. (2021) 137:2481–94. doi: 10.1182/blood.2020005627, PMID: 33171493 PMC7610744

[76] Buchta CM Bishop GA . TRAF5 negatively regulates TLR signaling in B lymphocytes. J Immunol. (2014) 192:145–50. doi: 10.4049/jimmunol.1301901, PMID: 24259503 PMC3872259

[77] Lan H Liu Y Liu J Wang X Guan Z Du J . Tumor-Associated Macrophages Promote Oxaliplatin Resistance via METTL3-Mediated m(6)A of TRAF5 and Necroptosis in Colorectal Cancer. Mol pharmaceutics. (2021) 18:1026–37. doi: 10.1021/acs.molpharmaceut.0c00961, PMID: 33555197

[78] Vadillo E Dorantes-Acosta E Pelayo R Schnoor M . T cell acute lymphoblastic leukemia (T-ALL): New insights into the cellular origins and infiltration mechanisms common and unique among hematologic Malignancies. Blood Rev. (2018) 32:36–51. doi: 10.1016/j.blre.2017.08.006, PMID: 28830639

[79] Chen L Shi Y Li J Yang X Li R Zhou X . LncRNA CDKN2B-AS1 contributes to tumorigenesis and chemoresistance in pediatric T-cell acute lymphoblastic leukemia through miR-335-3p/TRAF5 axis. Anticancer Drugs. (2020). doi: 10.1097/CAD.0000000000001001, PMID: 32976214

[80] Zhou R Mo W Wang S Zhou W Chen X Pan S . miR-141-3p and TRAF5 network contributes to the progression of T-cell acute lymphoblastic leukemia. Cell Transplant. (2019) 28:59S–65S. doi: 10.1177/0963689719887370, PMID: 31722554 PMC7016468

[81] Weston VJ Austen B Wei W Marston E Alvi A Lawson S . Apoptotic resistance to ionizing radiation in pediatric B-precursor acute lymphoblastic leukemia frequently involves increased NF-kappaB survival pathway signaling. Blood. (2004) 104:1465–73. doi: 10.1182/blood-2003-11-4039, PMID: 15142883

[82] Zhu S Jin J Gokhale S Lu AM Shan H Feng J . Genetic alterations of TRAF proteins in human cancers. Front Immunol. (2018) 9:2111. doi: 10.3389/fimmu.2018.02111, PMID: 30294322 PMC6158389

[83] Zou L Fang Y He W . TRAF7 inhibits glycolysis to potentiate growth inhibition and apoptosis of myeloid leukemia cells via regulating the KLF2-PFKFB3 axis. Mol Cell Probes. (2023) 69:101911. doi: 10.1016/j.mcp.2023.101911, PMID: 37003349

[84] Ding Q Wang Q Ren Y Zhu HQ Huang Z . MicroRNA-126 attenuates cell apoptosis by targeting TRAF7 in acute myeloid leukemia cells. Biochem Cell Biol = Biochimie biologie cellulaire. (2018) 96:840–6. doi: 10.1139/bcb-2018-0017, PMID: 29940130

[85] Dainichi T Matsumoto R Mostafa A Kabashima K . Immune control by TRAF6-mediated pathways of epithelial cells in the EIME (Epithelial immune microenvironment). Front Immunol. (2019) 10:1107. doi: 10.3389/fimmu.2019.01107, PMID: 31156649 PMC6532024

[86] Inoue J Gohda J Akiyama T . Characteristics and biological functions of TRAF6. Adv Exp Med Biol. (2007) 597:72–9. doi: 10.1007/978-0-387-70630-6_6, PMID: 17633018

[87] Kobayashi T Walsh MC Choi Y . The role of TRAF6 in signal transduction and the immune response. Microbes infection. (2004) 6:1333–8. doi: 10.1016/j.micinf.2004.09.001, PMID: 15555541

[88] Thakur N Sorrentino A Heldin CH Landström M . TGF-beta uses the E3-ligase TRAF6 to turn on the kinase TAK1 to kill prostate cancer cells. Future Oncol (London England). (2009) 5:1–3. doi: 10.2217/14796694.5.1.1, PMID: 19243289

[89] Choi Y . Role of TRAF6 in the immune system. Adv Exp Med Biol. (2005) 560:77–82. doi: 10.1007/0-387-24180-9_11, PMID: 15932023

[90] Qin Q Shan Z Xing L Jiang Y Li M Fan L . Synergistic effect of mesenchymal stem cell-derived extracellular vesicle and miR-137 alleviates autism-like behaviors by modulating the NF-κB pathway. J Transl Med. (2024) 22:446. doi: 10.1186/s12967-024-05257-w, PMID: 38741170 PMC11089771

[91] Jie XL Guo H Zhou GB . The E3 ubiquitin ligase TRAF6 controls CTLA-4 turnover and promotes T-cell-mediated antitumor immunity. Cell Mol Immunol. (2024) 21:97–9. doi: 10.1038/s41423-023-01105-x, PMID: 38082145 PMC10757707

[92] He Y Zou P Lu J Lu Y Yuan S Zheng X . CD4+ T-cell legumain deficiency attenuates hypertensive damage via preservation of TRAF6. Circ Res. (2024) 134:9–29. doi: 10.1161/CIRCRESAHA.123.322835, PMID: 38047378

[93] Wang J Shen S You J Wang Z Li Y Chen Y . PRMT6 facilitates EZH2 protein stability by inhibiting TRAF6-mediated ubiquitination degradation to promote glioblastoma cell invasion and migration. Cell Death Dis. (2024) 15:524. doi: 10.1038/s41419-024-06920-2, PMID: 39043634 PMC11266590

[94] Shen H Yuan J Tong D Chen B Yu E Chen G . Regulator of G protein signaling 16 restrains apoptosis in colorectal cancer through disrupting TRAF6-TAB2-TAK1-JNK/p38 MAPK signaling. Cell Death Dis. (2024) 15:438. doi: 10.1038/s41419-024-06803-6, PMID: 38906869 PMC11192724

[95] Giehler F Ostertag MS Sommermann T Weidl D Sterz KR Kutz H . Epstein-Barr virus-driven B cell lymphoma mediated by a direct LMP1-TRAF6 complex. Nat Commun. (2024) 15:414. doi: 10.1038/s41467-023-44455-w, PMID: 38195569 PMC10776578

[96] Li XM Yang Y Jiang FQ Hu G Wan S Yan WY . Histone lactylation inhibits RARγ expression in macrophages to promote colorectal tumorigenesis through activation of TRAF6-IL-6-STAT3 signaling. Cell Rep. (2024) 43:113688. doi: 10.1016/j.celrep.2024.113688, PMID: 38245869

[97] Daver NG Maiti A Kadia TM Vyas P Majeti R Wei AH . TP53-mutated myelodysplastic syndrome and acute myeloid leukemia: biology, current therapy, and future directions. Cancer discov. (2022) 12:2516–29. doi: 10.1158/2159-8290.CD-22-0332, PMID: 36218325 PMC9627130

[98] Tran TM Rao DS . RNA binding proteins in MLL-rearranged leukemia. Exp Hematol Oncol. (2022) 11:80. doi: 10.1186/s40164-022-00343-5, PMID: 36307883 PMC9615162

[99] Zhong X Ma H . Targeting CD38 for acute leukemia. Front Oncol. (2022) 12:1007783. doi: 10.3389/fonc.2022.1007783, PMID: 36313735 PMC9597453

[100] Seo W Silwal P Song IC Jo EK . The dual role of autophagy in acute myeloid leukemia. J Hematol Oncol. (2022) 15:51. doi: 10.1186/s13045-022-01262-y, PMID: 35526025 PMC9077970

[101] Long NA Golla U Sharma A Claxton DF . Acute myeloid leukemia stem cells: origin, characteristics, and clinical implications. Stem Cell Rev Rep. (2022) 18:1211–26. doi: 10.1007/s12015-021-10308-6, PMID: 35050458 PMC10942736

[102] Thielemans N Demeyer S Mentens N Gielen O Provost S Cools J . TAL1 cooperates with PI3K/AKT pathway activation in T-cell acute lymphoblastic leukemia. Haematologica. (2022) 107:2304–17. doi: 10.3324/haematol.2021.279718, PMID: 35354248 PMC9521226

[103] Wang Y Krivtsov AV Sinha AU North TE Goessling W Feng Z . The Wnt/beta-catenin pathway is required for the development of leukemia stem cells in AML. Sci (New York NY). (2010) 327:1650–3. doi: 10.1126/science.1186624, PMID: 20339075 PMC3084586

[104] Giambra V Jenkins CE Lam SH Hoofd C Belmonte M Wang X . Leukemia stem cells in T-ALL require active Hif1α and Wnt signaling. Blood. (2015) 125:3917–27. doi: 10.1182/blood-2014-10-609370, PMID: 25934477 PMC4548498

[105] Lu D Choi MY Yu J Castro JE Kipps TJ Carson DA . Salinomycin inhibits Wnt signaling and selectively induces apoptosis in chronic lymphocytic leukemia cells. Proc Natl Acad Sci U States A. (2011) 108:13253–7. doi: 10.1073/pnas.1110431108, PMID: 21788521 PMC3156152

[106] Yahata T Ibrahim AA Hirano KI Muguruma Y Naka K Hozumi K . Targeting of plasminogen activator inhibitor-1 activity promotes elimination of chronic myeloid leukemia stem cells. Haematologica. (2021) 106:483–94. doi: 10.3324/haematol.2019.230227, PMID: 32001531 PMC7849585

[107] Schrappe M Reiter A Zimmermann M Harbott J Ludwig WD Henze G . Long-term results of four consecutive trials in childhood ALL performed by the ALL-BFM study group from 1981 to 1995. Berlin-Frankfurt-Münster Leukemia. (2000) 14:2205–22. doi: 10.1038/sj.leu.2401973, PMID: 11187912

[108] Geng D Zheng L Srivastava R Velasco-Gonzalez C Riker A Markovic SN . Amplifying TLR-MyD88 signals within tumor-specific T cells enhances antitumor activity to suboptimal levels of weakly immunogenic tumor antigens. Cancer Res. (2010) 70:7442–54. doi: 10.1158/0008-5472.CAN-10-0247, PMID: 20807806 PMC3463001

[109] Geng D Zheng L Srivastava R Asprodites N Velasco-Gonzalez C Davila E . When Toll-like receptor and T-cell receptor signals collide: a mechanism for enhanced CD8 T-cell effector function. Blood. (2010) 116:3494–504. doi: 10.1182/blood-2010-02-268169, PMID: 20696947 PMC2981476

[110] LaRosa DF Stumhofer JS Gelman AE Rahman AH Taylor DK Hunter CA . T cell expression of MyD88 is required for resistance to Toxoplasma gondii. Proc Natl Acad Sci U States A. (2008) 105:3855–60. doi: 10.1073/pnas.0706663105, PMID: 18308927 PMC2268781

[111] Quigley M Martinez J Huang X Yang Y . A critical role for direct TLR2-MyD88 signaling in CD8 T-cell clonal expansion and memory formation following vaccinia viral infection. Blood. (2009) 113:2256–64. doi: 10.1182/blood-2008-03-148809, PMID: 18948575 PMC2652371

[112] Li Z Younger K Gartenhaus R Joseph AM Hu F Baer MR . Inhibition of IRAK1/4 sensitizes T cell acute lymphoblastic leukemia to chemotherapies. J Clin Invest. (2015) 125:1081–97. doi: 10.1172/JCI75821, PMID: 25642772 PMC4362243

[113] Calvi LM Link DC . The hematopoietic stem cell niche in homeostasis and disease. Blood. (2015) 126:2443–51. doi: 10.1182/blood-2015-07-533588, PMID: 26468230 PMC4661168

[114] Sánchez-Aguilera A Méndez-Ferrer S . The hematopoietic stem-cell niche in health and leukemia. Cell Mol Life sciences: CMLS. (2017) 74:579–90. doi: 10.1007/s00018-016-2306-y, PMID: 27436341 PMC5272896

[115] Chen YL Tang C Zhang MY Huang WL Xu Y Sun HY . Blocking ATM-dependent NF-kappaB pathway overcomes niche protection and improves chemotherapy response in acute lymphoblastic leukemia. Leukemia. (2019) 33:2365–78. doi: 10.1038/s41375-019-0458-0, PMID: 30940905

[116] Lewis BP Burge CB Bartel DP . Conserved seed pairing, often flanked by adenosines, indicates that thousands of human genes are microRNA targets. Cell. (2005) 120:15–20. doi: 10.1016/j.cell.2004.12.035, PMID: 15652477

[117] Calin GA Croce CM . MicroRNA signatures in human cancers. Nat Rev Cancer. (2006) 6:857–66. doi: 10.1038/nrc1997, PMID: 17060945

[118] Beltran JA Peek J Chang SL . Expression and regulation of the mu opioid peptide receptor in TPA-differentiated HL-60 promyelocytic leukemia cells. Int immunopharmacol. (2006) 6:1331–40. doi: 10.1016/j.intimp.2006.03.017, PMID: 16782547

[119] Tse AK Wan CK Shen XL Zhu GY Cheung HY Yang M . 1,25-dihydroxyvitamin D3 induces biphasic NF-kappaB responses during HL-60 leukemia cells differentiation through protein induction and PI3K/Akt-dependent phosphorylation/degradation of IkappaB. Exp Cell Res. (2007) 313:1722–34. doi: 10.1016/j.yexcr.2007.02.022, PMID: 17397830

[120] Chang CP Su YC Hu CW Lei HY . TLR2-dependent selective autophagy regulates NF-κB lysosomal degradation in hepatoma-derived M2 macrophage differentiation. Cell Death differentiation. (2013) 20:515–23. doi: 10.1038/cdd.2012.146, PMID: 23175187 PMC3569990

[121] Chang CP Su YC Lee PH Lei HY . Targeting NFKB by autophagy to polarize hepatoma-associated macrophage differentiation. Autophagy. (2013) 9:619–21. doi: 10.4161/auto.23546, PMID: 23360732 PMC3627680

[122] Fang J Barker B Bolanos L Liu X Jerez A Makishima H . Myeloid Malignancies with chromosome 5q deletions acquire a dependency on an intrachromosomal NF-kappaB gene network. Cell Rep. (2014) 8:1328–38. doi: 10.1016/j.celrep.2014.07.062, PMID: 25199827 PMC4237069

[123] Yuan W Sun Q Jiang Y Zhang X Chen L Xie C . MiR-146a affects the alteration in myeloid differentiation induced by hydroquinone in human CD34(+) hematopoietic progenitor cells and HL-60 cells. Toxicol Res (Camb). (2016) 5:848–58. doi: 10.1039/C5TX00419E, PMID: 30090394 PMC6061933

[124] Xiang M Birkbak NJ Vafaizadeh V Walker SR Yeh JE Liu S . STAT3 induction of miR-146b forms a feedback loop to inhibit the NF-κB to IL-6 signaling axis and STAT3-driven cancer phenotypes. Sci Signaling. (2014) 7:ra11. doi: 10.1126/scisignal.2004497, PMID: 24473196 PMC4233120

[125] Li Y Wang Y Yu L Sun C Cheng D Yu S . miR-146b-5p inhibits glioma migration and invasion by targeting MMP16. Cancer letters. (2013) 339:260–9. doi: 10.1016/j.canlet.2013.06.018, PMID: 23796692

[126] Burger ML Xue L Sun Y Kang C Winoto A . Premalignant PTEN-deficient thymocytes activate microRNAs miR-146a and miR-146b as a cellular defense against Malignant transformation. Blood. (2014) 123:4089–100. doi: 10.1182/blood-2013-11-539411, PMID: 24735967 PMC4073325

[127] Mitsumura T Ito Y Chiba T Matsushima T Kurimoto R Tanaka Y . Ablation of miR-146b in mice causes hematopoietic Malignancy. Blood Adv. (2018) 2:3483–91. doi: 10.1182/bloodadvances.2018017954, PMID: 30530754 PMC6290096

[128] Garzon R Marcucci G Croce CM . Targeting microRNAs in cancer: rationale, strategies and challenges. Nat Rev Drug discov. (2010) 9:775–89. doi: 10.1038/nrd3179, PMID: 20885409 PMC3904431

[129] Lieberman J . Tapping the RNA world for therapeutics. Nat Struct Mol Biol. (2018) 25:357–64. doi: 10.1038/s41594-018-0054-4, PMID: 29662218 PMC6052442

[130] Ling H Fabbri M Calin GA . MicroRNAs and other non-coding RNAs as targets for anticancer drug development. Nat Rev Drug discov. (2013) 12:847–65. doi: 10.1038/nrd4140, PMID: 24172333 PMC4548803

[131] Chen Y Gao DY Huang L . *In vivo* delivery of miRNAs for cancer therapy: challenges and strategies. Advanced Drug delivery Rev. (2015) 81:128–41. doi: 10.1016/j.addr.2014.05.009, PMID: 24859533 PMC5009470

[132] Rupaimoole R Slack FJ . MicroRNA therapeutics: towards a new era for the management of cancer and other diseases. Nat Rev Drug discov. (2017) 16:203–22. doi: 10.1038/nrd.2016.246, PMID: 28209991

[133] Su YL Wang X Mann M Adamus TP Wang D Moreira DF . Myeloid cell-targeted miR-146a mimic inhibits NF-κB-driven inflammation and leukemia progression *in vivo*. Blood. (2020) 135:167–80. doi: 10.1182/blood.2019002045, PMID: 31805184 PMC6966933

[134] Zou Q Tan S Yang Z Zhan Q Jin H Xian J . NPM1 mutant mediated PML delocalization and stabilization enhances autophagy and cell survival in leukemic cells. Theranostics. (2017) 7:2289–304. doi: 10.7150/thno.19439, PMID: 28740552 PMC5505061

[135] Tang Y Tao Y Wang L Yang L Jing Y Jiang X . NPM1 mutant maintains ULK1 protein stability via TRAF6-dependent ubiquitination to promote autophagic cell survival in leukemia. FASEB J. (2021) 35:e21192. doi: 10.1096/fj.201903183RRR, PMID: 33201521

[136] Daver N Schlenk RF Russell NH Levis MJ . Targeting FLT3 mutations in AML: review of current knowledge and evidence. Leukemia. (2019) 33:299–312. doi: 10.1038/s41375-018-0357-9, PMID: 30651634 PMC6365380

[137] Daver N Venugopal S Ravandi F . FLT3 mutated acute myeloid leukemia: 2021 treatment algorithm. Blood Cancer J. (2021) 11:104. doi: 10.1038/s41408-021-00495-3, PMID: 34045454 PMC8159924

[138] Wu M Li C Zhu X . FLT3 inhibitors in acute myeloid leukemia. J Hematol Oncol. (2018) 11:133. doi: 10.1186/s13045-018-0675-4, PMID: 30514344 PMC6280371

[139] Burchert A . Maintenance therapy for FLT3-ITD-mutated acute myeloid leukemia. Haematologica. (2021) 106:664–70. doi: 10.3324/haematol.2019.240747, PMID: 33472354 PMC7927878

[140] Schnetzke U Fischer M Kuhn AK Spies-Weisshart B Zirm E Hochhaus A . The E3 ubiquitin ligase TRAF6 inhibits LPS-induced AKT activation in FLT3-ITD-positive MV4–11 AML cells. J Cancer Res Clin Oncol. (2013) 139:605–15. doi: 10.1007/s00432-012-1362-4, PMID: 23263202 PMC11824562

[141] Sutheesophon K Nishimura N Kobayashi Y Furukawa Y Kawano M Itoh K . Involvement of the tumor necrosis factor (TNF)/TNF receptor system in leukemic cell apoptosis induced by histone deacetylase inhibitor depsipeptide (FK228). J Cell Physiol. (2005) 203:387–97. doi: 10.1002/jcp.20235, PMID: 15515013

[142] Mardis ER Ding L Dooling DJ Larson DE McLellan MD Chen K . Recurring mutations found by sequencing an acute myeloid leukemia genome. New Engl J Med. (2009) 361:1058–66. doi: 10.1056/NEJMoa0903840, PMID: 19657110 PMC3201812

[143] Alexandrov LB Nik-Zainal S Wedge DC Aparicio SA Behjati S Biankin AV . Signatures of mutational processes in human cancer. Nature. (2013) 500:415–21. doi: 10.1038/nature12477, PMID: 23945592 PMC3776390

[144] Welch JS Ley TJ Link DC Miller CA Larson DE Koboldt DC . The origin and evolution of mutations in acute myeloid leukemia. Cell. (2012) 150:264–78. doi: 10.1016/j.cell.2012.06.023, PMID: 22817890 PMC3407563

[145] Muto T Guillamot M Yeung J Fang J Bennett J Nadorp B . TRAF6 functions as a tumor suppressor in myeloid Malignancies by directly targeting MYC oncogenic activity. Cell Stem Cell. (2022) 29:298–314.e9. doi: 10.1016/j.stem.2021.12.007, PMID: 35045331 PMC8822959

[146] Ravandi F O’Brien S . Immune defects in patients with chronic lymphocytic leukemia. Cancer immunology immunother: CII. (2006) 55:197–209. doi: 10.1007/s00262-005-0015-8, PMID: 16025268 PMC11029864

[147] Molica S Levato D Levato L . Infections in chronic lymphocytic leukemia. Analysis of incidence as a function of length of follow-up. Haematologica. (1993) 78:374–7., PMID: 8175032

[148] Itälä M Helenius H Nikoskelainen J Remes K . Infections and serum IgG levels in patients with chronic lymphocytic leukemia. Eur J haematol. (1992) 48:266–70. doi: 10.1111/j.1600-0609.1992.tb01805.x, PMID: 1644158

[149] López-Collazo E del Fresno C . Pathophysiology of endotoxin tolerance: mechanisms and clinical consequences. Crit Care (London England). (2013) 17:242. doi: 10.1186/cc13110, PMID: 24229432 PMC4059412

[150] Jurado-Camino T Cordoba R Esteban-Burgos L Hernandez-Jimenez E Toledano V Hernandez-Rivas JA . Chronic lymphocytic leukemia: a paradigm of innate immune cross-tolerance. J Immunol. (2015) 194:719–27. doi: 10.4049/jimmunol.1402272, PMID: 25505275

[151] Amarante-Mendes GP Rana A Datoguia TS Hamerschlak N Brumatti G . BCR-ABL1 tyrosine kinase complex signaling transduction: challenges to overcome resistance in chronic myeloid leukemia. Pharmaceutics. (2022) 14:215. doi: 10.3390/pharmaceutics14010215, PMID: 35057108 PMC8780254

[152] Westermann J Bullinger L . Precision medicine in myeloid Malignancies. Semin Cancer Biol. (2022) 84:153–69. doi: 10.1016/j.semcancer.2021.03.034, PMID: 33895273

[153] Poudel G Tolland MG Hughes TP Pagani IS . Mechanisms of resistance and implications for treatment strategies in chronic myeloid leukaemia. Cancers. (2022) 14:3300. doi: 10.3390/cancers14143300, PMID: 35884363 PMC9317051

[154] Teng M Luskin MR Cowan-Jacob SW Ding Q Fabbro D Gray NS . The dawn of allosteric BCR-ABL1 drugs: from a phenotypic screening hit to an approved drug. J medicinal Chem. (2022) 65:7581–94. doi: 10.1021/acs.jmedchem.2c00373, PMID: 35609336

[155] Han SH Korm S Han YG Choi SY Kim SH Chung HJ . GCA links TRAF6-ULK1-dependent autophagy activation in resistant chronic myeloid leukemia. Autophagy. (2019) 15:2076–90. doi: 10.1080/15548627.2019.1596492, PMID: 30929559 PMC6844495

[156] Fang J Muto T Kleppe M Bolanos LC Hueneman KM Walker CS . TRAF6 mediates basal activation of NF-κB necessary for hematopoietic stem cell homeostasis. Cell Rep. (2018) 22:1250–62. doi: 10.1016/j.celrep.2018.01.013, PMID: 29386112 PMC5971064

[157] Varney ME Niederkorn M Konno H Matsumura T Gohda J Yoshida N . Loss of Tifab, a del(5q) MDS gene, alters hematopoiesis through derepression of Toll-like receptor-TRAF6 signaling. J Exp Med. (2015) 212:1967–85. doi: 10.1084/jem.20141898, PMID: 26458771 PMC4612089

